# Timing of Complete Revascularization in Patients with STEMI and Multivessel Disease: A Systematic Review and Meta-Analysis

**DOI:** 10.31083/j.rcm2402058

**Published:** 2023-02-10

**Authors:** Giuseppe Panuccio, Nadia Salerno, Salvatore De Rosa, Daniele Torella

**Affiliations:** ^1^Department of Medical and Surgical Sciences, Magna Graecia University, 88100 Catanzaro, Italy; ^2^Department of Experimental and Clinical Medicine, Magna Graecia University, 88100 Catanzaro, Italy

**Keywords:** revascularization, multivessel, coronary artery disease, STEMI, PCI

## Abstract

**Background:**

About half of patients with ST-segment Elevation Myocardial Infarction (STEMI) have multivessel coronary artery disease (MVD). Our aim was to provide a quantitative comparison of single-stage complete revascularization during the index revascularization versus deferred staged complete revascularization in STEMI patients with MVD.

**Methods:**

All studies evaluating patients with STEMI and MVD were included. The primary endpoint was a composite of all-cause death, myocardial infarction and repeat revascularization. Secondary endpoints were cardiovascular death, acute kidney injury and trial defined major bleeding.

**Results:**

Eight studies and 2256 patients with STEMI and MVD were included. No difference was evident in the rate of the primary composite endpoint among the study group (Risk Ratio 0.95; 95% CI 0.71–1.27, *p* = 0.74), while meta-regression showed a significant interaction with drug eluting stent (DES) use (Coefficient –0.005; 95% CI –0.01 to –0.001; *p* = 0.007). Higher rates of cardiovascular (CV) death were found in the immediate complete revascularization group (5.0% vs 2.6%; Risk Ratio 0.39; 95% CI 0.25–0.62; *p *< 0.01).

**Conclusions:**

Our analysis documented similar clinical outcomes with either single-stage immediate complete revascularization and delayed staged complete revascularization. Secondary analyses suggest that an increase in cardiovascular death might be expected with single-stage percutaneous coronary intervention (PCI). While new randomized trials on the topic are ongoing, revascularization can be personalized and guided by the acute clinical setting, patients’-related factors and workflow logistics.

## 1. Introduction

Acute ST-segment Elevation Myocardial Infarction (STEMI) is a life-threatening disorder bringing along a high morbidity load. Hence, it represents a challenge to patients and the society, despite advances in treatment [1]. Percutaneous coronary intervention (PCI) represents a cornerstone for the treatment of patients with Acute Myocardial Infarction (AMI). Above 50% of AMI patients are estimated to have multivessel coronary artery disease (MVD), which is often associated with poorer outcomes [2, 3, 4]. Several randomized trials, including the PRAMI (Preventive Angioplasty in Myocardial Infarction) [5], CvLPRIT (Complete versus Lesion-only Primary PCI trial) [6], DANAMI-3-PRIMULTI (Third Danish Study of Optimal Acute Treatment of Patients with ST-segment Elevation Myocardial Infarction: Primary PCI in Multivessel Disease) [7], COMPARE-ACUTE (Comparison Between FFR Guided Revascularization Versus Conventional Strategy in Acute STEMI Patients With Multivessel Disease After Early PCI for STEMI) [8] and COMPLETE (Complete Versus Culprit-Only Revascularization Strategies to Treat Multivessel Disease After Early PCI for STEMI) [9] trials showed that complete coronary revascularization is superior to culprit-only PCI in reducing the risk of cardiovascular death or re-infarction, or ischemia-driven revascularization. Having confirmed that complete revascularization outperforms culprit-only PCI, a new dilemma has more recently arisen: what is the most effective timing for complete revascularization in MVD patients? Is deferred staged revascularization any different compared to immediate complete revascularization during the index STEMI procedure? A recent meta-analysis found worrisome signals of worse outcomes in AMI patients treated with MV-PCI during the index intervention in the context of cardiogenic shock [10]. However, the jury is still out on the optimal timing of complete revascularization in more hemodynamically stable patients [11, 12, 13].

Therefore, the aim of this global meta-analysis was to provide a quantitative comparison of two alternative complete revascularization strategies, namely immediate complete revascularization versus deferred staged complete revascularization in STEMI patients with MVD.

## 2. Methods

This meta-analysis was performed according to the Cochrane Collaboration and PRISMA guidelines [14, 15].

### 2.1 Research

Scientific literature was systematically searched for studies reporting on clinical outcomes for different strategies of complete revascularization in patients with STEMI. Articles were searched for on the following public databases: PubMed (https://pubmed.ncbi.nlm.nih.gov/) and ProQuest (https://www.proquest.com/index) until April 4th 2022. We used the following keywords: staged (pci or PTCA), multivessel.

### 2.2 Study Selection with Inclusion/Exclusion Criteria

Two co-authors (GP, SDR) independently assessed search records to identify eligible trials. Divergencies were resolved though discussion and consensus. Studies were eligible if they had all the pre-defined criteria for inclusion: (a) any clinical study in which different strategies of multivessel revascularization were adopted; (b) the clinical setting in which revascularization was performed was acute coronary syndrome (ACS)-STEMI; (c) clinical outcomes were reported. Exclusion criteria were: studies including patients with cardiogenic shock; editorial comments; case reports; review articles or meta-analysis; mean age of study population <18 years; case series of fewer than 10 patients included; clinical outcomes not reported; multivessel revascularization in other settings than STEMI (e.g., angina or any elective patient). The same co-authors were responsible for data extraction (GP, SDR). Baseline clinical characteristics were extracted to an excel worksheet, including age, gender, cardiovascular risk factors, infarct location, procedural characteristics such as Syntax score, number of treated lesions, number of stents implanted, type of stents used (bare metal stent (BMS) or drug eluting stent (DES) or other), in addition to outcomes data (short and long-term mortality, cardiovascular death, re-infarction, repeat revascularization acute kidney injury and trial defined major bleeding).

### 2.3 Outcomes

The primary analysis was based on the primary composite outcome of all cause death, myocardial infarction (MI) and repeat revascularization. Additionally, cardiovascular death, acute kidney injury and trial defined major bleeding (defined as Bleeding Academic Research Consortium (BARC) >2, the thrombolysis in myocardial infarction (TIMI) Major or Global Use of Strategies to Open Occluded Coronary Arteries (GUSTO) severe bleeding) were also analyzed as secondary endpoints.

### 2.4 Evaluation of Study Quality

Study quality was assessed by 2 co-authors (GP, SDR). Divergences were managed though discussion and agreement to a consensus. The risk of multiple form of bias were evaluated: confounding, selection, classification of therapeutic interventions, deviations, missing data, outcomes’ measurement, selection of the reported results, in accord to ROBINS-II tool [16].

### 2.5 Statistical Analysis

Continuous variables were synthesized as mean ± standard deviation, while discrete variables were expressed as percentages, as previously described [17]. Summary effect sizes were calculated using the random-effects model described by Mantel-Haenszel, and results were presented as Risk Ratios (RR) with 95% Confidence Intervals (95% CI). *p* values < 0.05 were considered significant. The number of patients needed to harm (NNH) was calculated as the inverse of the absolute risk reduction, rounded up to the nearest integer number.

Meta-analysis calculations were performed using OpenMetaAnalyst 10 (Brown University, Providence, Rhode Island, USA) and RevMan 5.4 (The Cochrane Collaboration, The Nordic Cochrane Centre, Copenhagen, Denmark). Meta regression analysis was performed using Comprehensive Meta-analysis Software (Biostat Inc.14 North Dean Street Englewood, NJ, USA), using the restricted maximum likelihood (reml), as previously described [18]. Study bias was appraised by graphical inspection of funnel plots and by Egger’s and Begg’s tests. Heterogeneity of studies was measured as the Inconsistency index ($I^{2}$) and tested using Cochran’s Q test.

The analysis protocol was registered in PROSPERO, international prospective register of systematic reviews (PROSPERO record ID = 359356).

## 3. Results

### 3.1 Selected Studies and Baseline Characteristics

**Fig. 1.**
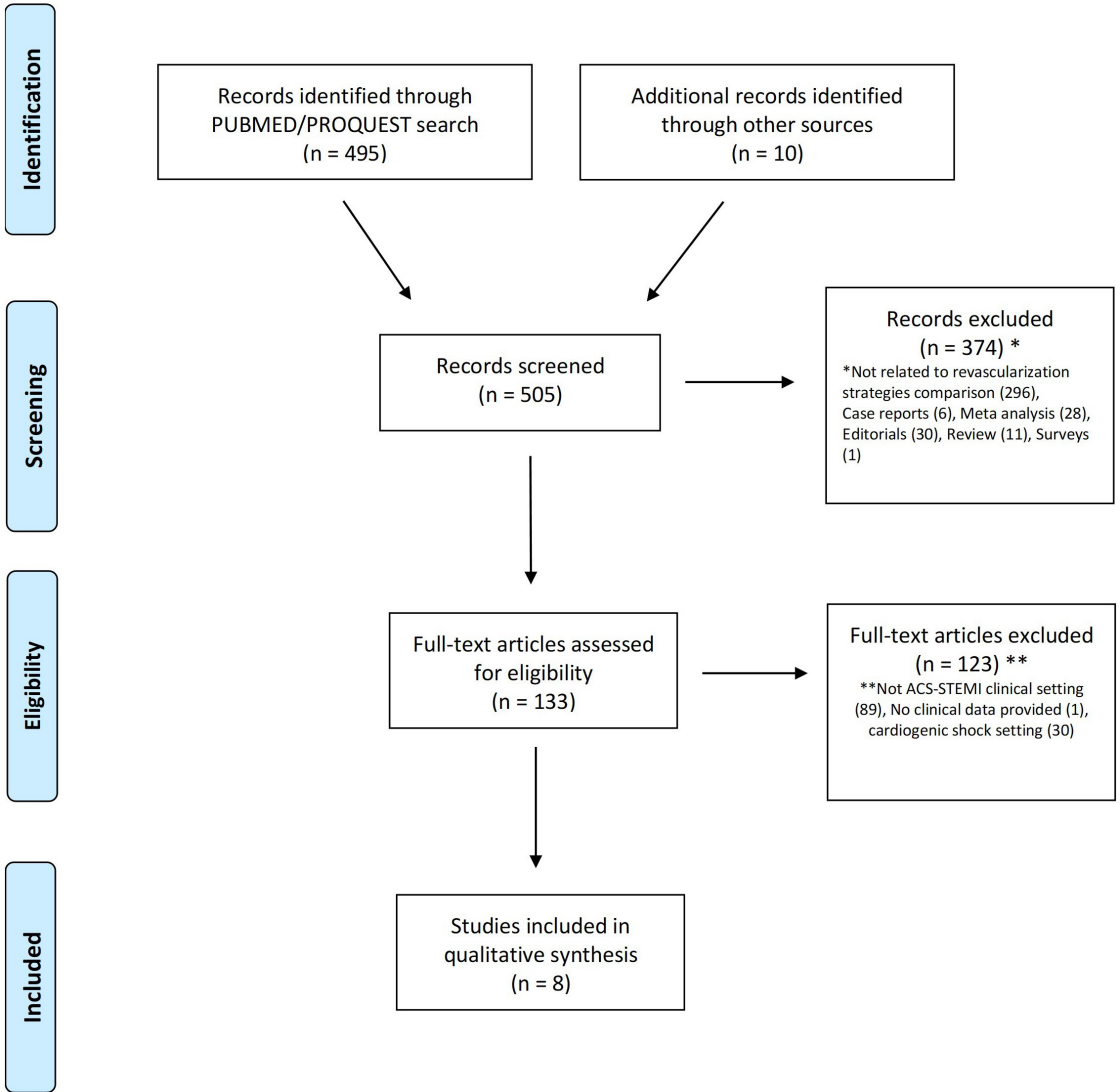
**Flow chart of included studies**.

From 505 studies identified, 8 studies [6, 19, 20, 21, 22, 23, 24, 25] (2256 patients with STEMI and multivessel disease) were included in this analysis (Fig. 1). Of the latter, 6 studies were Randomized Controlled Trials (RCTs) [6, 20, 21, 22, 23, 24], while the remaining 2 were non-randomized trials [19, 25]. Mean age was 61.5 ± 4.7 years. All patients were admitted for STEMI and most had a high cardiovascular risk profile. Studies baseline characteristics are provided in Table 1 (Ref. [6, 19, 20, 21, 22, 23, 24, 25]) while detailed information for single studies is shown in Table 2 (Ref. [6, 19, 20, 21, 22, 23, 24, 25]).

**Table 1.** **Study characteristics**.

Study	Corpus *et al*. [19]	Ochala *et al*. [20]	Politi *et al*. [21]	Maamoun *et al*. [22]	Kornowski *et al*. [23]	Gershlick *et al*. [6]	Taarasov *et al*. [24]	Kim *et al*. [25]
Year	2004	2004	2010	2011	2011	2015	2017	2020
Journal	American Heart Journal	Journal of Invasive Cardiology	Heart	The Egyptian Heart Journal	Journal of the American College of Cardiology	Journal of the American College of Cardiology	Interventional Cardiology	Catheter Cardiovasc Interv
Sample size	152	92	130	78	668	139	136	861
Study design	Nonrandomized Retrospective Single site	Randomized Prospective Multicentric	Randomized Prospective Single site	Randomized Prospective Single site	Randomized Prospective Multicentric	Randomized Prospective Multicentric	Randomized Prospective Single site	Non randomized Retrospective Multicentric
Timing of staged PCI	During index hospitalization	NR	56.8 ± 12.9 days	Within 7 days	30 days (range 6.0 to 51 days)	During index hospitalization	10.1 ± 5.1 days	3–6 days
Follow up (years)	1	0.5	2.5	1	1	1	1	3
Primary and secondary endpoint	(1) Death, re-infarction, target-vessel revascularization at 1 year after PCI. (2) Procedural complications (acute occlusion, perforation, stroke, major bleeding, vascular complications, urgent CABG).	(1) Absolute improvement of LVEF. (2) All cause death, AMI, urgent revascularization (including TVR).	(1) All-cause death, recurrent myocardial infarction, heart failure, and ischemia-driven revascularization within 12 months.	(1) Death, re-infarction, target-vessel revascularization at 1 year after PCI.	(1) Death, re-infarction, target-vessel revascularization for ischemia within 1 year.	(1) All-cause death, recurrent myocardial infarction (MI), heart failure, and ischemia-driven revascularization within 12 months.	(1) Death, re-infarction, target-vessel revascularization.	(1) All-cause mortality. (2) Cardiac mortality, recurrent myocardial infarction, repeat revascularization and stent thrombosis during a 3-year clinical follow-up.

PCI, Percutaneous Coronary Intervention; CABG, Coronary Artery Bypass Grafting; LVEF, Left Ventricle Ejection Fraction; AMI, Acute Myocardial Infarction; TVR, Target Vessel Revascularization; NR, Not reported.

**Table 2.** **Baseline patients’ characteristics**.

Study	Year	Age	Male sex (%)	Diabetes (%)	Hypertension (%)	3 vessel disease (%)	DES use (%)	CKD (%)	Femoral access (%)	Radial access (%)	Syntax Score	LVEF (%)	Anterior location/LAD IRA (%)
Ochala *et al*. [20]	2004	65.9	NR	34.9	49.9	NR	0.0	NR	NR	NR	NR	NR	45.6
Corpus *et al*. [19]	2004	61.5	NR	NR	NR	NR	0.0	NR	NR	NR	NR	NR	NR
Politi *et al*. [21]	2010	64.3	78.5	16.2	56.9	36.9	8.5	25.6	NR	NR	NR	45.6	47.7
Maamoun *et al*. [22]	2011	53.5	92.3	47.4	35.9	24.4	33.3	NR	NR	NR	NR	45.2	66.0
Kornowski *et al*. [23]	2011	62.9	80.3	16.9	56.4	NR	76.0	NR	NR	NR	NR	NR	37.9
Gershlick *et al*. [6]	2015	64.9	81.0	18.6	NR	22.7	93.3	NR	14.5	86.5	NR	NR	36.0
Taarasov *et al*. [24]	2017	58.9	66.9	22.1	91.9	46.3	100.0	NR	NR	NR	18.8	51.2	NR
Kim *et al*. [25]	2020	62.1	81.5	28.2	49.1	36.7	95.4	NR	76.7	23.3	NR	50.4	45.2

DES, Drug Eluting Stent; CKD, Chronic Kidney Disease; LVEF, Left Ventricle Ejection Fraction; LAD, Left Anterior Descending; IRA, Infarct Related Artery; NR, Not reported.

### 3.2 Primary Outcome

**Fig. 2.**
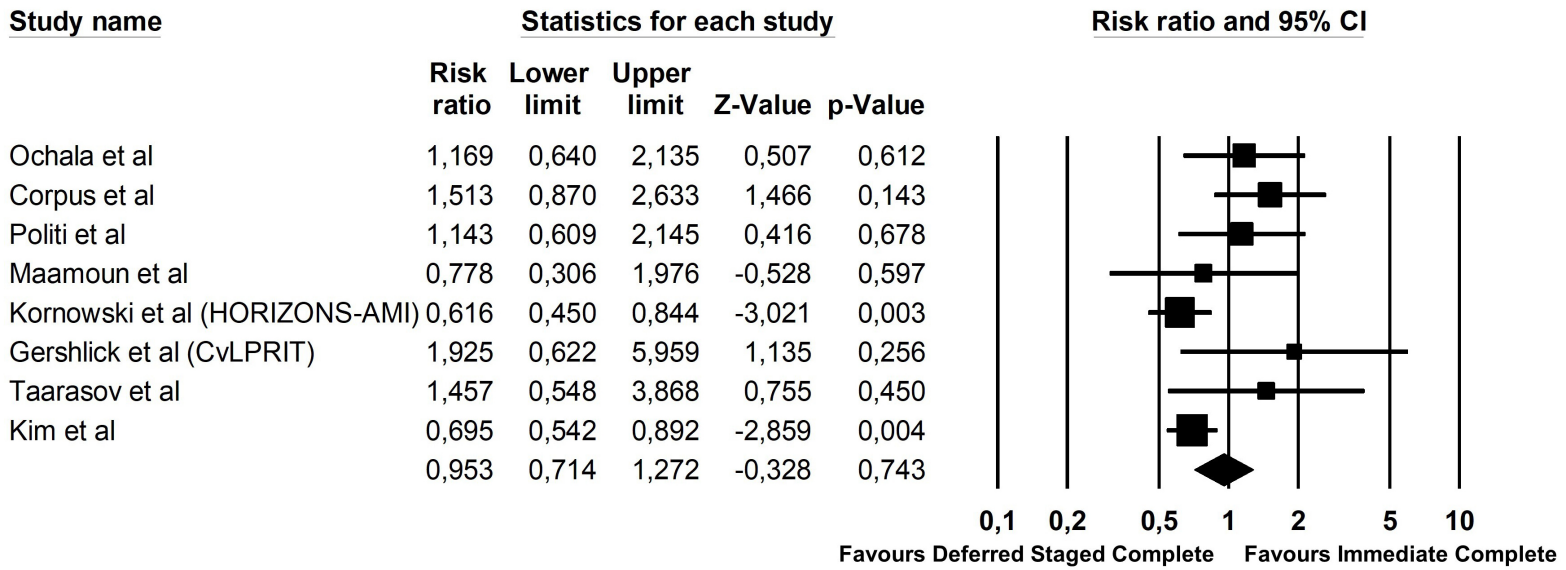
**Forest plot of the composite endpoint for STEMI and MVD**.

**Fig. 3.**
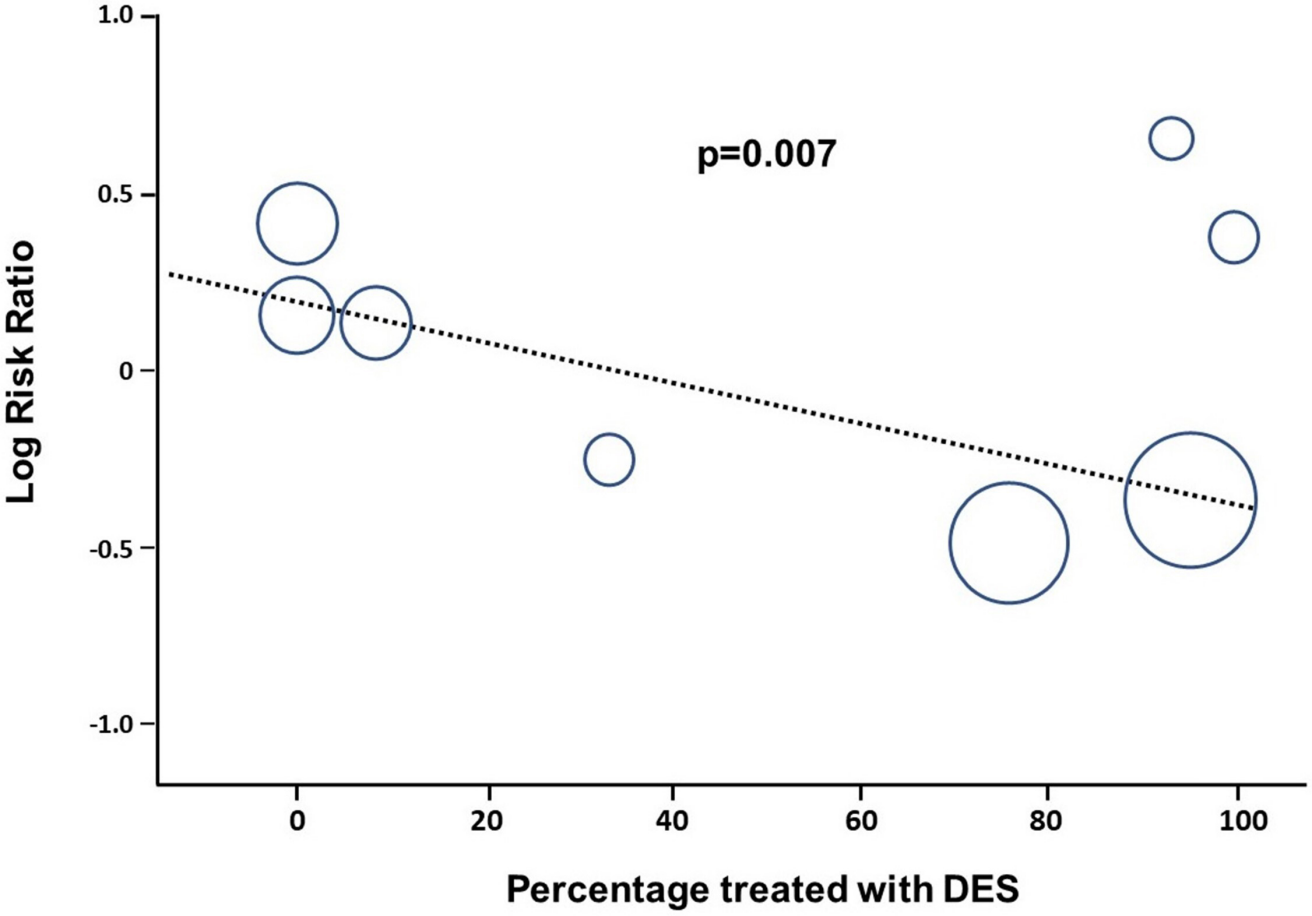
**Meta-regression analysis of DES use effect on the main composite main outcome**.

Of the 2256 patients included, 696 (17.9%) reached the primary endpoint. Of those, 272 patients (21.1%) reached the primary endpoint in the deferred staged complete revascularization group, while 220 patients (22.6%) reached the primary endpoint in the immediate complete revascularization group (Risk Ratio 0.95; 95% CI 0.71–1.27, *p* = 0.74, Fig. 2). Sensitivity analysis using the leave-one-out interaction method did not change the general outlook of the results, that remained consistent also across subgroups. Meta-regression analysis showed a significant interaction between DES use and the composite endpoint (*p* = 0.007, Fig. 3). On the contrary, no interaction was found with the time gap between the index and the staged procedure (*p* = 0.67) (**Supplementary Fig. 1**), nor with the proportion of anterior wall infarction (*p* = 0.89) (**Supplementary Fig. 2**).

### 3.3 Secondary Outcomes

**Fig. 4.**
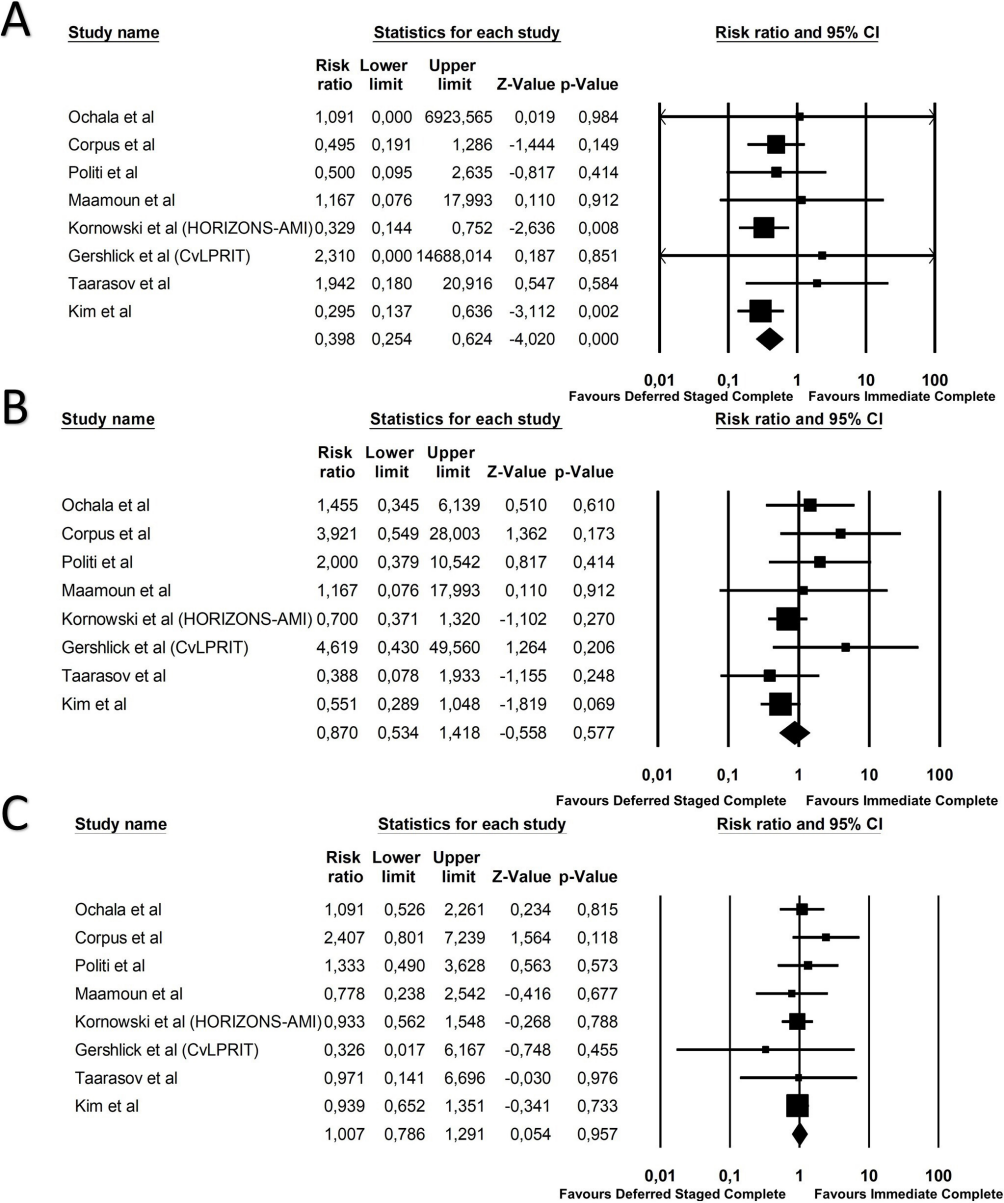
**Forest plot of secondary outcomes CV death (A), MI (B) and repeat revascularization (C)**.

CV death occurred on 34 patients in the deferred staged complete revascularization group and on 49 patients in the immediate complete revascularization group (Risk Ratio 0.40; 95% CI 0.25–0.62; *p *< 0.01, NNH = 42, Fig. 4A). At sensitivity analysis this difference was maintained after exclusion of the studies with low adoption of DES (Risk Ratio 0.35; 95% CI 0.20–0.60; *p *< 0.01, **Supplementary Fig. 3**). MI occurred in 66 patients in the deferred staged complete revascularization group and in 51 patients in the immediate complete revascularization group (Risk Ratio 0.87; 95% CI 0.53–1.41; *p* = 0.57, Fig. 4B). Repeat revascularization occurred in 152 patients in the deferred staged complete revascularization group and on 99 patients in the immediate complete revascularization group (Risk Ratio 1.09; 95% CI 0.78–1.29; *p* = 0.95, Fig. 4C). Acute kidney injury (AKI) occurred in 3 patients in the deferred staged complete revascularization group and on 3 patients in the immediate complete revascularization group (Risk Ratio 1.07; 95% CI 0.21–5.53; *p* = 0.93; **Supplementary Fig. 4**). Trial defined major bleeding occurred in 33 patients in the deferred staged complete revascularization group and on 27 patients in the immediate complete revascularization group (Risk Ratio 0.75; 95% CI 0.45–1.23; *p* = 0.25; **Supplementary Fig. 5**).

### 3.4 Study Quality 

Low to moderate risk of bias was found (**Supplementary Fig. 6**). Heterogeneity was low to moderate. Visual inspection of funnel plots did not demonstrate severe asymmetries. Results of both the Egger’s and Begg’s tests were in line with funnel plots (**Supplementary Figs. 7,8**).

## 4. Discussion

The optimal timing of non-culprit vessel revascularization in STEMI patients with MVD is still debated today [26]. After multiple trials comparing MV-PCI to culprit-only revascularization left no doubt about the superiority of complete revascularization, the dust has only partially settled: the debate is currently focused on the optimal timing of a complete revascularization. Current guidelines recommend staged PCI of significant non-infarct related artery stenoses in class I. PCI of non-infarct artery stenoses at the time of the primary revascularization is recommended in class IIb in selected hemodynamically stable patients with STEMI and low complexity multivessel disease [11]. The main analysis of the present study revealed no significant difference in the primary outcome between the treatment strategies. At the same time, and in line with recent findings for patients treated in the context of cardiogenic shock [10], we found a significant higher rates of cardiovascular death in patients undergoing single-staged MV-PCI in the index procedure, with a NNH of 42. Nevertheless, it should be noticed that several trials included in our analysis and referred to in current practice guidelines included a substantial proportion of patients who were not treated with DES. It is noteworthy to highlight that the degree of DES use significantly impacted on the outcomes in STEMI patients with MVD. Particularly, meta-regression analysis showed that larger DES use is significantly associated to a larger reduction of the primary composite endpoint of all cause death, MI and repeat revascularization (*p* = 0.007), suggesting that the use of DES might eventually flip the results in favor of single-stage immediate MV-PCI at the index procedure. However, these results are hypothesis-generating and not conclusive yet.

Some of the trials included were repeatedly criticized over the last years for signals of potential bias. In a post-hoc analysis of the CMR sub-study of CvLPRIT trial [27] was showed that in the study by Gershlick *et al*. [6], patients receiving staged revascularization had larger infarct scars and lower LV function, even after adjustment for relevant covariates, as compared with patients who underwent immediate complete revascularization, as reported by Khan *et al*. [28]. In the study by Kornowski *et al*. [23] there were significant differences in baseline characteristics between patients undergoing single-stage PCI and patients treated with staged PCI. Specifically, ejection fraction was significantly lower in the single stage PCI group as also reported in a letter to the editor by McCabe *et al*. [29]. In the study by Kim *et al*. [25], there was a significant difference in ejection fraction, radial access use and left main disease between staged and single stage PCI, and follow-up data provided reached 3 years from PCI. Also, in the subgroup analysis performed stratifying patients by means of the GRACE score, Kim *et al*. [25] showed that in patients with low to intermediate GRACE score, there were no significant differences in all cause death between the two revascularization approaches. However, at sensitivity analysis excluding those two studies from our analysis, results showed to be consistent with the absence of any significant difference between the two groups, even though a numerical trend emerged in favor of immediate (single-stage) complete revascularization for the composite endpoint of all cause death, repeat revascularization and MI (**Supplementary Fig. 9**).

Hybrid revascularization is an interesting approach to revascularization in patents with MVD. However, clinical evidence on this procedure is scanty, particularly regarding the timing between the surgical and the percutaneous procedures. Some hints and some caveats can be found from the limited literature available. The first, is the unexpected observation that hybrid revascularization gives its best in patients with Syntax scores ≤22 [30, 31]. More encouraging results come from a recent study that investigate one-stop complete hybrid revascularization showed good results also with patients with higher Syntax scores [32]. Nevertheless, it should be mentioned that no comparator arm with hybrid revascularization on different timing was present in this study. In addition, a one-stage complete hybrid revascularization in the acute setting still encounters resistance for technical reasons [32].

Several randomized controlled trials MULTISTARS AMI trial (NCT03135275), the FULL REVASC trial (NCT02862119), the SAFE STEMI for Seniors trial (NCT02939976), the FIRE trial (NCT03772743) and the FRAME-AMI trial (NCT027155189) are currently ongoing, and they will provide relevant pieces of information on this debated topic. Meanwhile, as both alternatives have a similarly efficacy profile in, the revascularization strategy can be personalized considering all procedural and patient-related factors. In particular, complete revascularization can be deferred especially in patients with renal dysfunction or when a substantial amount of contrast volume was already used to treat the culprit, in case of no-reflow/slow-flow after revascularization of the IRA, in presence of intermediate stenoses or when there is concern of overestimation of the angiographical severity of non-culprit lesions while single-stage complete revascularization could be reserved to patients with simple CAD anatomy, where a complete revascularization might be achieved with no much hassle and without substantial prolongation of procedural time or relevant increase in the amount of contrast medium, or in patients with difficult vascular access to avoid risks associated with a second percutaneous procedure.

## 5. Limitations

As usually happens with interventional trials adopting percutaneous treatments, the continuous and fast development of clinical strategies, interventional techniques and materials often renders study results outdated once they are published. Two of the studies included date back to 2004 [19, 20]. Consequently, not all materials and techniques adopted represent the current state of the art, which might limit applicability of our results to contemporary patients. This meta-analysis included retrospective studies, introducing a risk for selection bias. Also, there was a heterogeneous follow up length between studies. Nevertheless, sensitivity analysis showed that exclusion of retrospective studies and of studies with longest follow up from the analysis did not change the general results outlook (**Supplementary Fig. 10**). Another limitation of this meta-analysis is the total number of patients included. In fact, the studies that investigated this topic included a limited number of patients. In fact, a small portion of the studies dealing with the timing of complete revascularization compared immediate complete revascularization with deferred staged complete revascularization. Ongoing randomized trials will shed a light on this relevant topic. Not all studies included data on all secondary endpoints. Specifically, the study by Gershlick *et al*. [6], did not report data on CV death, MI and repeat revascularization. In addition, only few studies reported the average Syntax score of the patients, therefore we could not perform a subgroup analysis by this variable.

## 6. Conclusions

Multiple studies have compared culprit-only versus complete coronary revascularization in STEMI patients with multivessel disease [5, 6, 7, 8, 9]. However, no conclusive evidence is available on the optimal timing of complete revascularization. Our analysis documented similar clinical outcomes with either single-stage immediate complete revascularization and delayed staged complete revascularization. However, the higher incidence of cardiovascular death in the immediate complete revascularization sounds an alarm bell and should be further clarified. While ongoing randomized trials are expected to shed new light on this relevant topic, choices should be personalized to patients’ profiles and guided by the clinical context and workflow logistics.

## Data Availability

The datasets used and/or analyzed during the current study are available from the corresponding author on reasonable request.

## Supplementary Material

Supplementary material associated with this article can be found, in the online version, at https://doi.org/10.31083/j.rcm2402058.
