# Supplementary material for: Timing of Complete Revascularization in Patients with STEMI and Multivessel Disease: A Systematic Review and Meta-Analysis
Source: Rev Cardiovasc Med. 2023 Feb 10;24(2):58. doi: 10.31083/j.rcm2402058 (PMC11273107; doi:10.31083/j.rcm2402058)
Supplement: Supplementary file 1 [file 2153-8174-24-2-058-s1.zip › PRISMA_2020_abstract_checklist.pdf]

PRISMA 2020 for Abstracts Checklist

Section and Topic	Item #	Checklist item	Reported (Yes/No)
**TITLE**			
Title	1	Optimal timing of revascularization in patients with STEMI and multivessel disease: a systematic review and meta-analysis	Yes
**BACKGROUND**			
Objectives	2	To provide a quantitative comparison of single-stage complete revascularization during the index revascularization versus deferred staged complete revascularization in STEMI patients with MVD.	Yes
**METHODS**			
Eligibility criteria	3	a) any clinical study in which different strategies of multivessel revascularization were adopted; b) the clinical setting in which revascularization was performed was ACS-STEMI; c) clinical outcomes were reported.	No
Information sources	4	Scientific literature was searched for on the following public databases: PubMed (https://pubmed.ncbi.nlm.nih.gov/) and ProQuest (https://www.proquest.com/index) until April 4th 2022). We used the following keywords: staged, (pci or PTCA), multivessel.	No
Risk of bias	5	We evaluated the risk of bias (low, moderate, serious) for confounding, selection of participants, classification of interventions, deviation from intended intervention, missing data, measurement outcomes, selection of the reported results in accord to ROBINS-II tool.	No
Synthesis of results	6	Continuous variables are reported as mean and standard deviation. Categorical variables are expressed as percentages. Cumulative effect sizes were calculated according to a random-effects model by Mantel-Haenszel, and results presented as Risk Ratios (RR). 95% Confidence Intervals (95% CI) were provided for all outcomes. The number of patients needed to harm (NNH) was calculated as the inverse of the absolute risk reduction, rounded up to the nearest integer number.	Yes
**RESULTS**			
Included studies	7	10 studies (3867 patients with STEMI and multivessel disease) were included in this analysis. Of the latter, 7 studies were Randomized Controlled Trials (RCTs), while the remaining 3 were non-randomized trials.	Yes
Synthesis of results	8	From 505 studies identified, 10 studies (3867 patients with STEMI and multivessel disease) were included in this analysis. Of the latter, 7 studies were Randomized Controlled Trials (RCTs), while the remaining 3 were non-randomized trials. Of the 3867 patients included, 696 (17.9%) reached the primary endpoint. Of those, 362 patients reached the primary endpoint in the deferred staged complete revascularization group, while 334 patients reached the primary endpoint in the immediate complete revascularization group (Risk Ratio 0.93; 95% CI 0.74-1.17, p=0.52); Sensitivity analysis using the leave-one-out interaction method did not change the general outlook of the results, that remained consistent also across subgroups. Meta-regression analysis showed a significant interaction between DES use and the composite endpoint (p=0.007).CV death occurred on 75 patients in the deferred staged complete revascularization group and on 101 patients in the immediate complete revascularization group (Risk Ratio 0.57; 95% CI 0.34-0.94; p=0.03, NNH=59). Sensitivity analysis showed that this difference was lost after exclusion of the studies with low adoption of DES, as this effect was completely lost after exclusion of the studies with lowest DES adoption (Risk Ratio	Yes

PRISMA 2020 for Abstracts Checklist

Section and Topic	Item #	Checklist item	Reported (Yes/No)
		0.59; 95% CI 0.29-1.24; p=0.16). MI occurred in 83 patients in the deferred staged complete revascularization group and in 77 patients in the immediate complete revascularization group (Risk Ratio 0.82; 95% CI 0.57-1.19; p=0.30). Repeat revascularization occurred in 183 patients in the deferred staged complete revascularization group and on 136 patients in the immediate complete revascularization group (Risk Ratio 1.01; 95% CI 0.81-1.26; p=0.91). AKI occurred in 3 patients in the deferred staged complete revascularization group and on 3 patients in the immediate complete revascularization group (Risk Ratio 1.08; 95% CI 0.21-5.55; p=0.92). Trial defined major bleeding occurred in 78 patients in the deferred staged complete revascularization group and on 70 patients in the immediate complete revascularization group (Risk Ratio 0.82; 95% CI 0.60-1.12; p=0.21).	
**DISCUSSION**			
Limitations of evidence	9	This meta-analysis included retrospective studies, introducing a risk for selection bias. Also, there was a heterogeneous follow up length between studies. Nevertheless, sensitivity analysis showed that exclusion of retrospective studies and of studies with longest follow up from the analysis did not change the general results outlook.	Yes
Interpretation	10	Our analysis documented similar clinical outcomes with either single-stage immediate complete revascularization and delayed staged complete revascularization, especially when DES are used. While ongoing randomized trials are expected to shed new light on this relevant topic, choices should be personalized to patients' profile and guided by the clinical context and workflow logistics.	Yes
**OTHER**			
Funding	11	None.	
Registration	12	This meta-analysis and its protocol have been registered on the PROSPERO international prospective register of systematic reviews (PROSPERO record ID=359356).	

From: Page MJ, McKenzie JE, Bossuyt PM, Boutron I, Hoffmann TC, Mulrow CD, et al. The PRISMA 2020 statement: an updated guideline for reporting systematic reviews. BMJ 2021;372:n71. doi: 10.1136/bmj.n71

For more information, visit: http://www.prisma-statement.org/
