# Supplementary material for: Timing of Complete Revascularization in Patients with STEMI and Multivessel Disease: A Systematic Review and Meta-Analysis
Source: Rev Cardiovasc Med. 2023 Feb 10;24(2):58. doi: 10.31083/j.rcm2402058 (PMC11273107; doi:10.31083/j.rcm2402058)
Supplement: Supplementary file 1 [file 2153-8174-24-2-058-s1.zip › PRISMA_2020_checklist.pdf]

PRISMA 2020 Checklist

Section and Topic	Item #	Checklist item	Location where item is reported
**TITLE**			
Title	1	Optimal timing of revascularization in patients with STEMI and multivessel disease: a systematic review and meta-analysis.	Title Page
**ABSTRACT**			
Abstract	2	See the PRISMA 2020 for Abstracts checklist.	
**INTRODUCTION**			
Rationale	3	Recent randomized trials showed that complete revascularization is superior to culprit-lesion-only PCI in ACS-STEMI and multivessel-disease (MVD) The jury is still out on the optimal timing of complete revascularization.	Introduction
Objectives	4	To provide a quantitative comparison of two alternative revascularization strategies, namely immediate complete revascularization versus deferred staged complete revascularization in STEMI patients with MVD.	Introduction
**METHODS**			
Eligibility criteria	5	a) any clinical study in which different strategies of multivessel revascularization were adopted; b) the clinical setting in which revascularization was performed was ACS-STEMI; c) clinical outcomes were reported.	Methods
Information sources	6	Scientific literature was searched for on the following public databases: PubMed (https://pubmed.ncbi.nlm.nih.gov/) and ProQuest (https://www.proquest.com/index) until April 4th 2022.	Methods
Search strategy	7	The following keywords were used: staged, (pci or PTCA), multivessel.	Methods
Selection process	8	Two reviewers (GP, SDR) independently screened search records to identify eligible trials. Divergencies were resolved though discussion on study methodology until consensus was reached. Studies were selected if they fulfilled all the pre-defined inclusion criteria reported.	Methods
Data collection process	9	Data extraction was performed by two independent reviewers (GP, SDR), with divergences resolved by consensus. Baseline characteristics of the patients included were extracted to an excel worksheet, including age, gender, cardiovascular risk factors, infarct location, procedural characteristics, in addition to outcomes data.	Methods
Data items	10a	The outcomes for which data were sought were: all cause death, myocardial infarction (MI) and repeat revascularization. Additionally, cardiovascular death, acute kidney injury and trial defined major bleeding (defined as BARC>2, TIMI Major or GUSTO severe bleeding) were also analyzed as secondary endpoints. All results were compatible with each outcome domain in each study.	Methods
	10b	All other variables for which data were sought were: age, gender, cardiovascular risk factors, infarct location, procedural characteristics (Syntax score, number of treated lesions, number of stents implanted, type of stents used (BMS or DES or other), study design, timing of staged-PCI, follow up (expressed in years). No data were missing regarding the primary outcome, if there were any missing or unclear information regarding other variables, they were reported and those studies were not included in meta-regression analyses.	Methods
Study risk of bias assessment	11	The risk of bias (low, moderate, serious) was evaluated for confounding, selection of participants, classification of interventions, deviation from intended intervention, missing data, measurement outcomes, selection of the reported results in accord to ROBINS-II tool. Risk of bias evaluation was performed by two independent reviewers (GP, SDR), with divergences resolved by consensus.	Methods
Effect measures	12	For each outcome the effect measure(s) used in the synthesis or presentation of results were risk ratios with 95% Confidence Intervals (95% CI) provided for all outcomes. The number of patients needed to harm (NNH) was calculated as the inverse of the absolute risk reduction, rounded up to the nearest integer number.	Methods
Synthesis methods	13a	The processes used to decide which studies were eligible for each synthesis were if they fulfilled the following criteria: a) any clinical study in which different strategies of multivessel revascularization were adopted; b) the clinical setting in which revascularization was performed was ACS-STEMI; c) clinical outcomes were reported.	Methods
	13b	No methods were required to prepare the data for presentation or synthesis, such as handling of missing summary statistics, or data conversions.	Methods
	13c	The methods used to tabulate or visually display results of the studies and syntheses have been tables and forest plots.	Methods

Section and Topic	Item #	Checklist item	Location where item is reported
	13d	Cumulative effect sizes were calculated according to a random-effects model by Mantel-Haenszel, and results presented as Risk Ratios (RR). Heterogeneity of studies was measured by means of the Inconsistency index (I2) and tested using Cochran's Q test. Software packages used were OpenMetaAnalyst 10 (Brown University, Providence, Rhode Island) and RevMan 5.4 (The Cochrane Collaboration, The Nordic Cochrane Centre, Copenhagen, Denmark). Meta regression analysis was performed using Comprehensive Meta-analysis Software (Biostat Inc.14 North Dean Street Englewood, NJ, USA).	Methods
	13e	Meta-regression analysis was used for evaluating the effect of DES use on the primary endpoint.	Methods
	13f	Sensitivity analyses were performed to a) excluding retrospective studies and studies with longest follow; b) excluding studies with low adoption of DES; c) excluding the studies from Kornowski et al. and Kim et al.	Methods
Reporting bias assessment	14	Risk of bias was evaluated in accord to ROBINS-II tool.	Methods
Certainty assessment	15	The Grading of Recommendations Assessment, Development and Evaluation (GRADE) approach was used to assess the certainty in the body of evidence for all outcomes.	
**RESULTS**			
Study selection	16a	A flow diagram was used to describe the results of the search and selection process, from the number of records identified in the search to the number of studies included in the meta-analysis.	Figure 1
	16b	Some studies might appear to meet the inclusion criteria, but they were excluded, because they did not report the clinical outcomes related to this meta-analysis.	Figure 1
Study characteristics	17	Ten (10) studies (3867 patients with STEMI and multivessel disease) were included in this analysis. Of the latter, 7 studies were Randomized Controlled Trials (RCTs), while the remaining 3 were non-randomized trials.	Results
Risk of bias in studies	18	Evaluation of possible biases related to the included studies demonstrated a low to moderate risk of bias; traffic light plots were used for showing the evaluation of bias.	Supplementary Figure 4
Results of individual studies	19	For all outcomes of each study: (a) summary statistics for each group was provided; (b) cumulative effect sizes were calculated according to a random-effects model by Mantel-Haenszel, and results presented as Risk Ratios (RR). 95% Confidence Intervals (95% CI) were provided for all outcomes; c) forest plots and scatter plots were used to provide detailed information on the analyses.	Methods
Results of syntheses	20a	From 10 studies included, 7 were Randomized Controlled Trials (RCT). Six of them were considered to have low risk of bias, 1 of them had moderate risk of bias. The 3 non-randomized trials included were considered to have high-risk of bias.	Supplementary Figure 4
	20b	From 505 studies identified, 10 studies (3867 patients with STEMI and multivessel disease) were included in this analysis. Of the latter, 7 studies were Randomized Controlled Trials (RCTs), while the remaining 3 were non-randomized trials. Of the 3867 patients included, 696 (17.9%) reached the primary endpoint. Of those, 362 patients reached the primary endpoint in the deferred staged complete revascularization group, while 334 patients reached the primary endpoint in the immediate complete revascularization group (Risk Ratio 0.93; 95% CI 0.74-1.17, p=0.52); Sensitivity analysis using the leave-one-out interaction method did not change the general outlook of the results, that remained consistent also across subgroups. Meta-regression analysis showed a significant interaction between DES use and the composite endpoint (p=0.007).CV death occurred on 75 patients in the deferred staged complete revascularization group and on 101 patients in the immediate complete revascularization group (Risk Ratio 0.57; 95% CI 0.34-0.94; p=0.03, NNH=59). Sensitivity analysis showed that this difference was lost after exclusion of the studies with low adoption of DES, as this effect was completely lost after exclusion of the studies with lowest DES adoption (Risk Ratio 0.59; 95% CI 0.29-1.24; p=0.16). MI occurred in 83 patients in the deferred staged complete revascularization group and in 77 patients in the immediate complete revascularization group (Risk Ratio 0.82; 95% CI 0.57-1.19; p=0.30). Repeat revascularization occurred in 183 patients in the deferred staged complete revascularization group and on 136 patients in the immediate complete revascularization group (Risk Ratio 1.01; 95% CI 0.81-1.26; p=0.91). AKI occurred in 3 patients in the deferred staged complete revascularization group and on 3 patients in the immediate complete revascularization group (Risk Ratio 1.08; 95% CI 0.21-5.55; p=0.92). Trial defined major bleeding occurred in 78 patients in the deferred staged complete revascularization group and on 70 patients in	Results; Figure 2-4; Supplementary Figure 1-2-3-7-8

Section and Topic	Item #	Checklist item	Location where item is reported
		the immediate complete revascularization group (Risk Ratio 0.82; 95% CI 0.60-1.12; p=0.21).	
	20c	Heterogeneity was low to moderate. Graphical evaluation of the funnel plots did not demonstrate severe asymmetries as confirmed by the Egger's and Begg's tests.	Supplementary Figure 5.
	20d	Sensitivity analysis using the leave-one-out interaction method did not change the general outlook of the results, that remained consistent also across subgroups. Sensitivity analysis showed that the difference in CV death between the two revascularization strategies was lost after exclusion of the studies with low adoption of DES, as this effect was completely lost after exclusion of the studies with lowest DES adoption. Sensitivity analysis excluding the studies from Karnowski et al. and Kim et al. showed to be consistent with the absence of any significant difference between the two groups, even though a numerical trend emerged in favor of immediate (single-stage) complete revascularization for the composite endpoint of all cause death, repeat revascularization and MI.	Supplementary Figure 1-7-8.
Reporting biases	21	Not all studies included data on all secondary endpoints. Specifically, the study by Gershlick et al, did not report data on CV death, MI and repeat revascularization. Also, in the study by Rathod et al, no information on the percentage of DES use was provided, so we could not include it in the meta-regression analysis for the effect of DES use on the primary endpoint. In addition, only few studies reported the average Syntax score of the patients, therefore we could not perform a subgroup analysis by this variable. Sensitivity analysis using the leave-one-out interaction method did not change the general outlook of the results, that remained consistent also across subgroups.	Limitations
Certainty of evidence	22	The Grading of Recommendations Assessment, Development and Evaluation (GRADE) approach was used to assess the certainty in the body of evidence for every outcome.	
**DISCUSSION**			
Discussion	23a	Our analysis documented similar clinical outcomes with either single-stage immediate complete revascularization and delayed staged complete revascularization, especially when DES are used.	Conclusions
	23b	This meta-analysis included retrospective studies, introducing a risk for selection bias. Nevertheless, sensitivity analysis showed that exclusion of retrospective studies and of studies with longest follow up from the analysis did not change the general results outlook.	Limitations
	23c	There was a heterogeneous follow up length between studies. Nevertheless, sensitivity analysis showed that exclusion of retrospective studies and of studies with longest follow up from the analysis did not change the general results outlook.	Limitations
	23d	While ongoing randomized trials are expected to shed new light on this relevant topic, choices should be personalized to patients' profile and guided by the clinical context and workflow logistics.	Conclusions
**OTHER INFORMATION**			
Registration and protocol	24a	This meta-analysis and its protocol have been registered on the PROSPERO international prospective register of systematic reviews (PROSPERO record ID=359356).	Methods
	24b	https://www.crd.york.ac.uk/prospero/	
	24c	No amendments to information have been made at registration or in the protocol.	
Support	25	No financial or non-financial support for the review was given.	
Competing interests	26	The of review authors have no competing interests.	
Availability of data, code and other materials	27	The template of the Prisma Flow Diagram can be found at https://prisma-statement.org/prismastatement/flowdiagram.aspx.	

From: Page MJ, McKenzie JE, Bossuyt PM, Boutron I, Hoffmann TC, Mulrow CD, et al. The PRISMA 2020 statement: an updated guideline for reporting systematic reviews. BMJ 2021;372:n71. doi: 10.1136/bmj.n71
