# Supplementary material for: Timing of Complete Revascularization in Patients with STEMI and Multivessel Disease: A Systematic Review and Meta-Analysis
Source: Rev Cardiovasc Med. 2023 Feb 10;24(2):58. doi: 10.31083/j.rcm2402058 (PMC11273107; doi:10.31083/j.rcm2402058)
Supplement: Supplementary file 1 [file 2153-8174-24-2-058-s1.zip › Supplementary data - R1-final.pptx]

## Slide 1
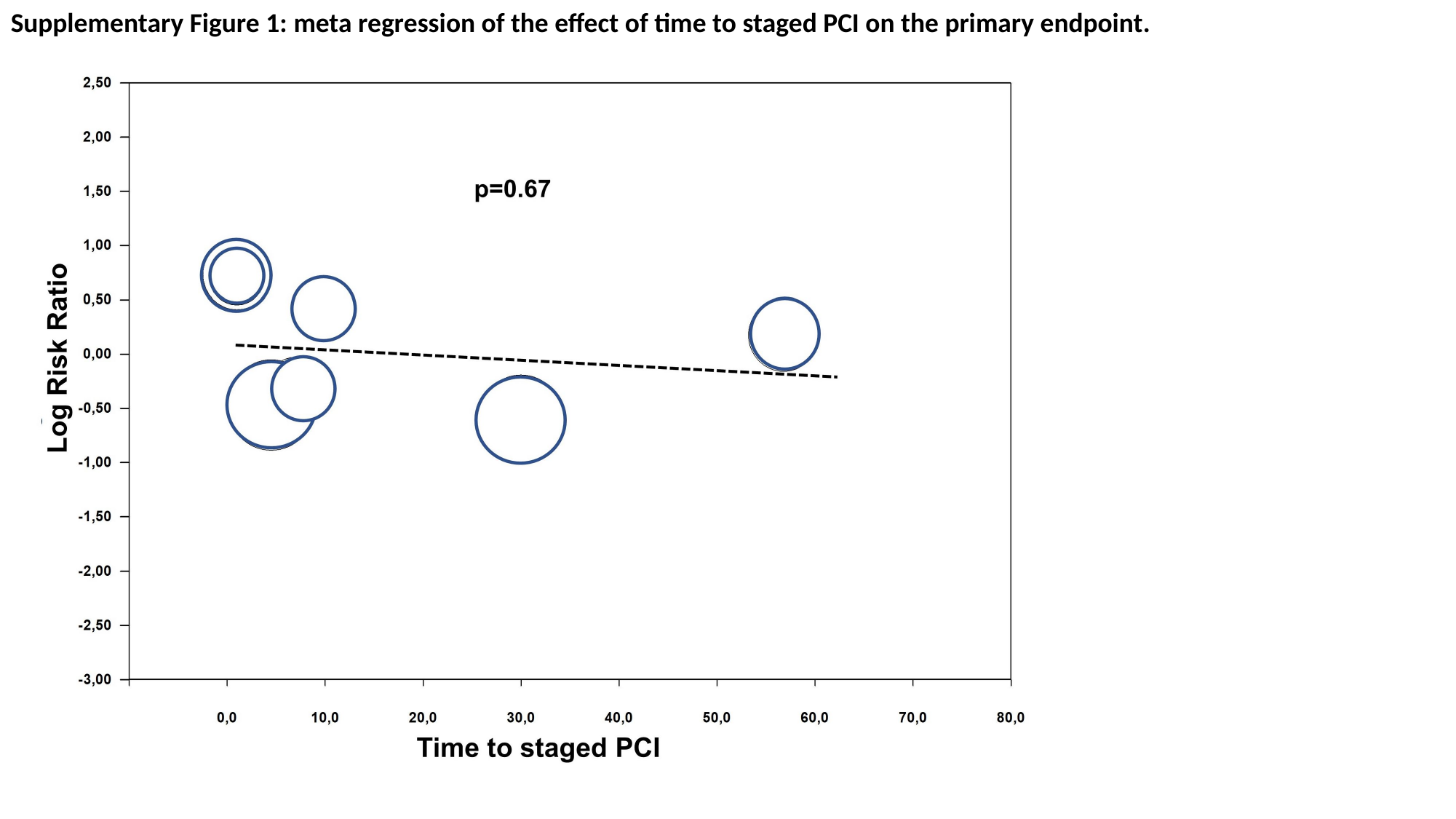

Supplementary Figure 1: meta regression of the effect of time to staged PCI on the primary endpoint.

## Slide 2
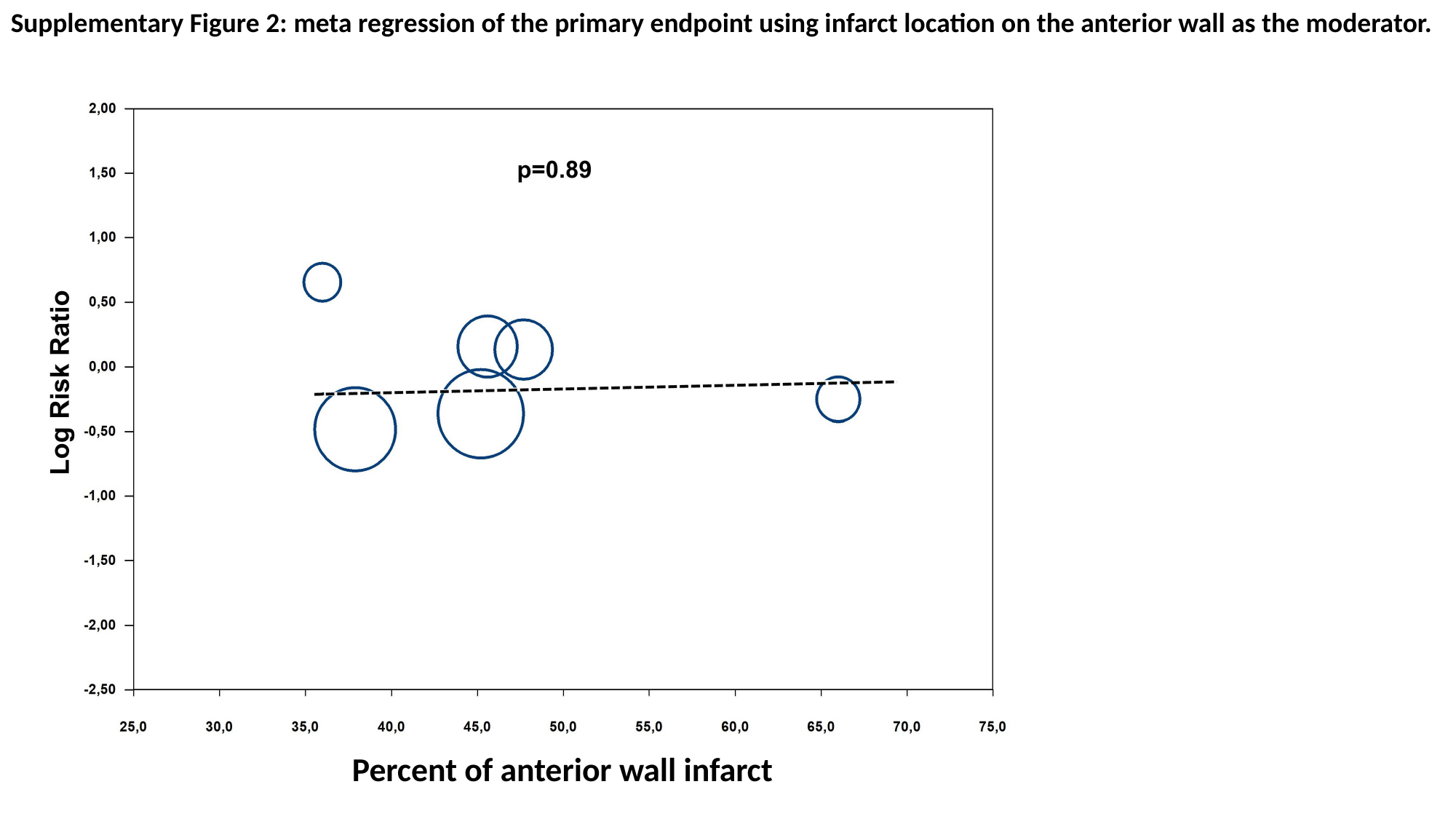

Supplementary Figure 2: meta regression of the primary endpoint using infarct location on the anterior wall as the moderator.
Percent of anterior wall infarct

## Slide 3
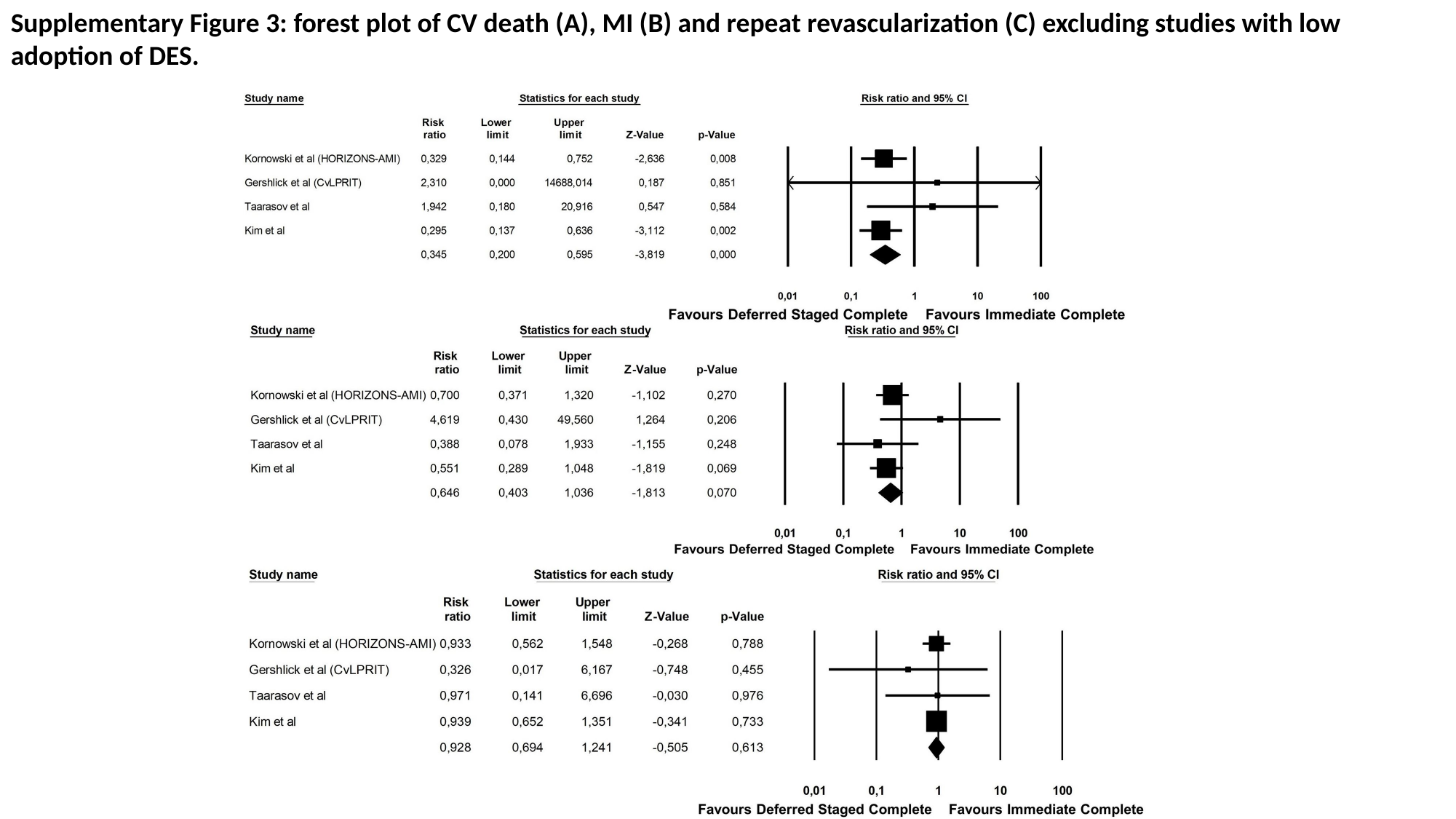

Supplementary Figure 3: forest plot of CV death (A), MI (B) and repeat revascularization (C) excluding studies with low adoption of DES.

## Slide 4
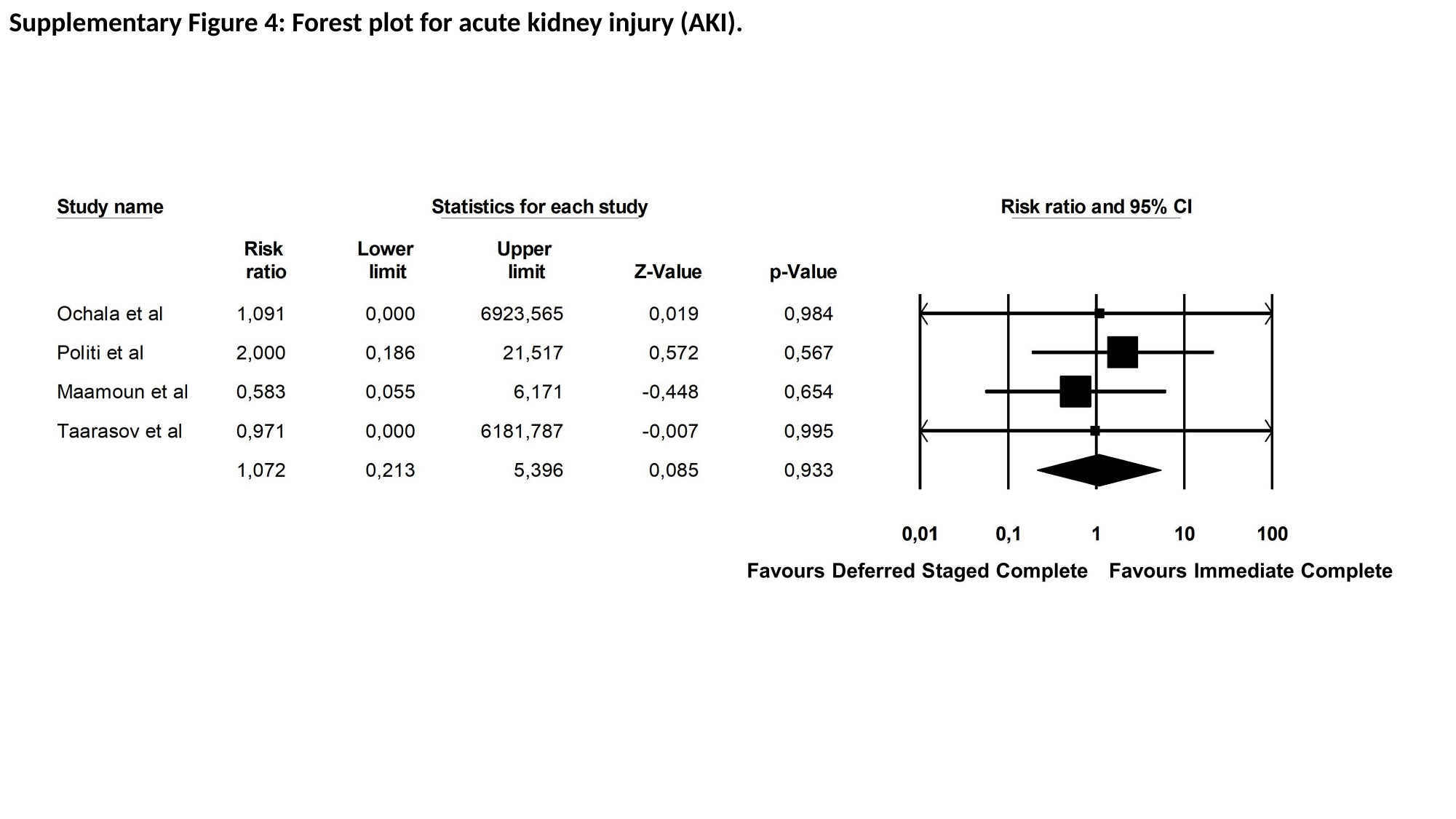

Supplementary Figure 4: Forest plot for acute kidney injury (AKI).

## Slide 5
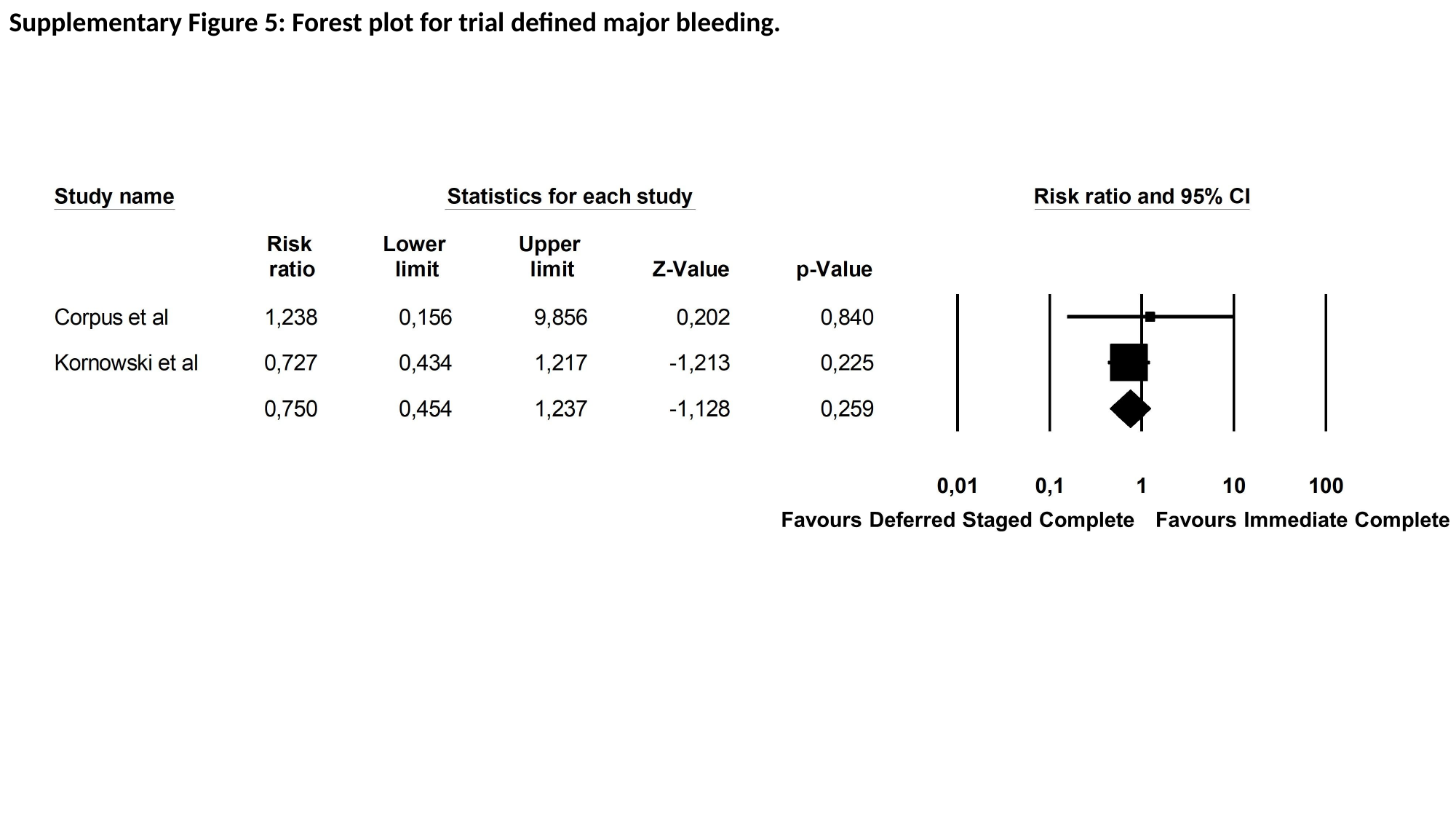

Supplementary Figure 5: Forest plot for trial defined major bleeding.

## Slide 6
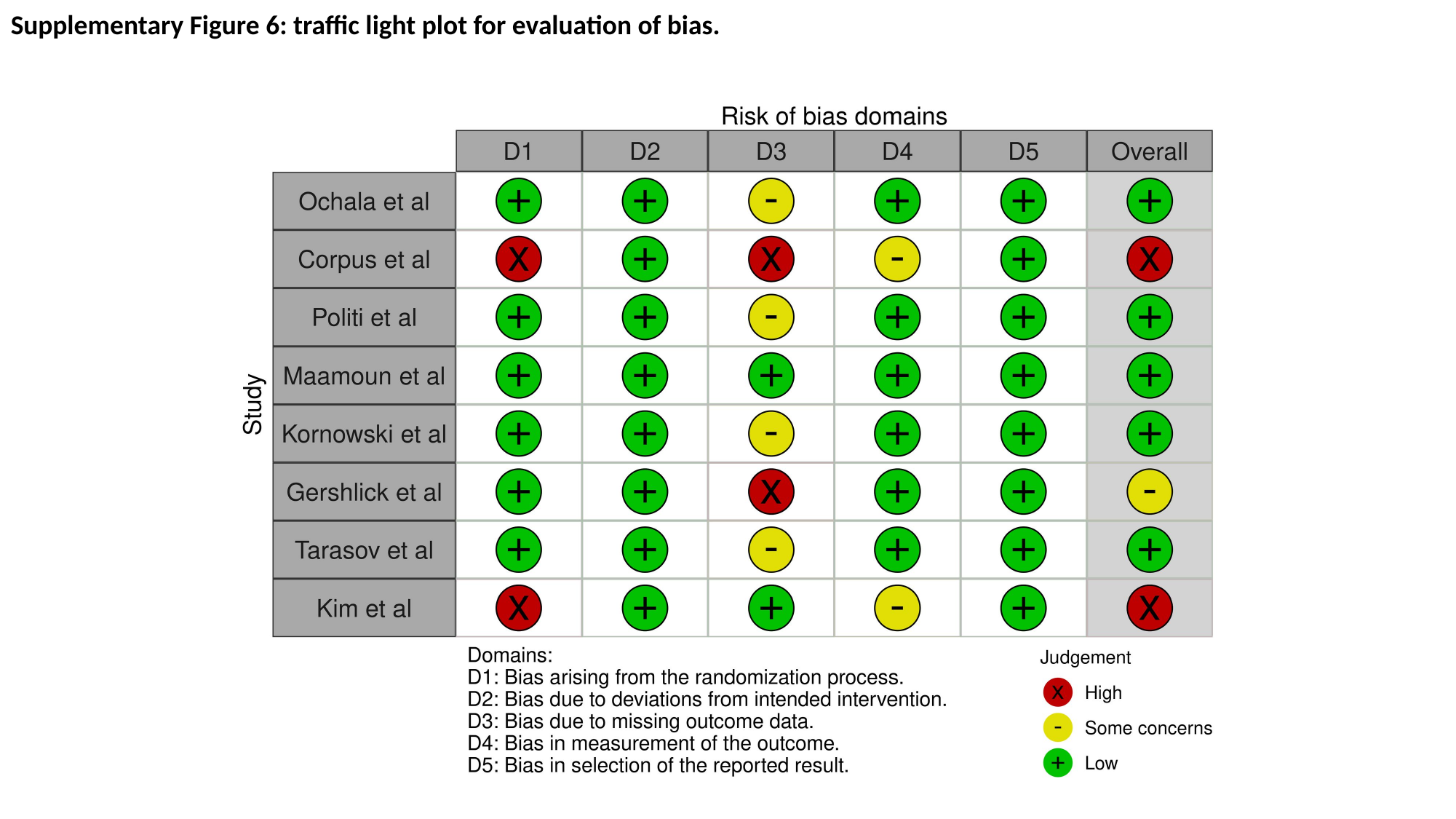

Supplementary Figure 6: traffic light plot for evaluation of bias.

## Slide 7
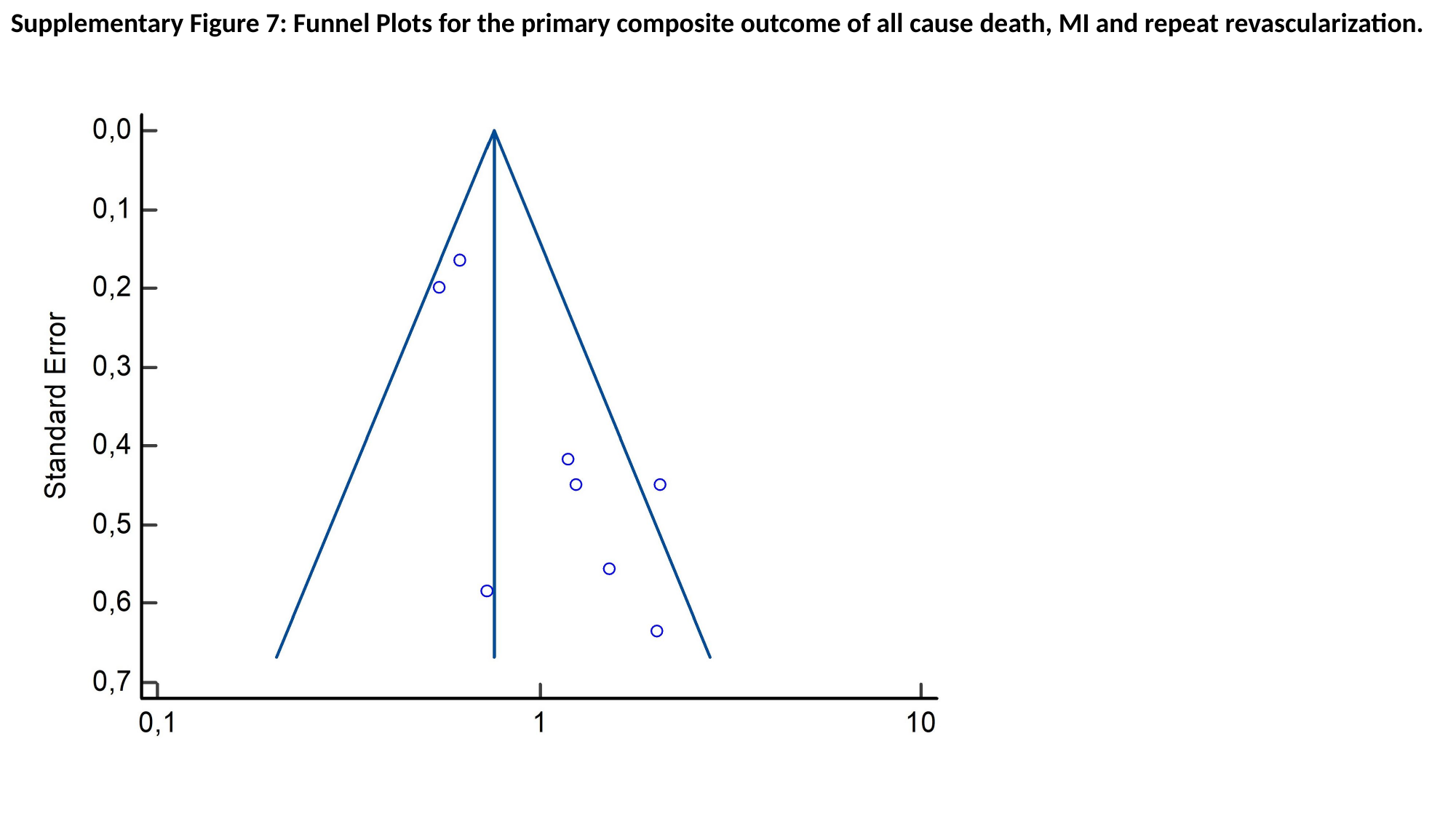

Supplementary Figure 7: Funnel Plots for the primary composite outcome of all cause death, MI and repeat revascularization.

## Slide 8
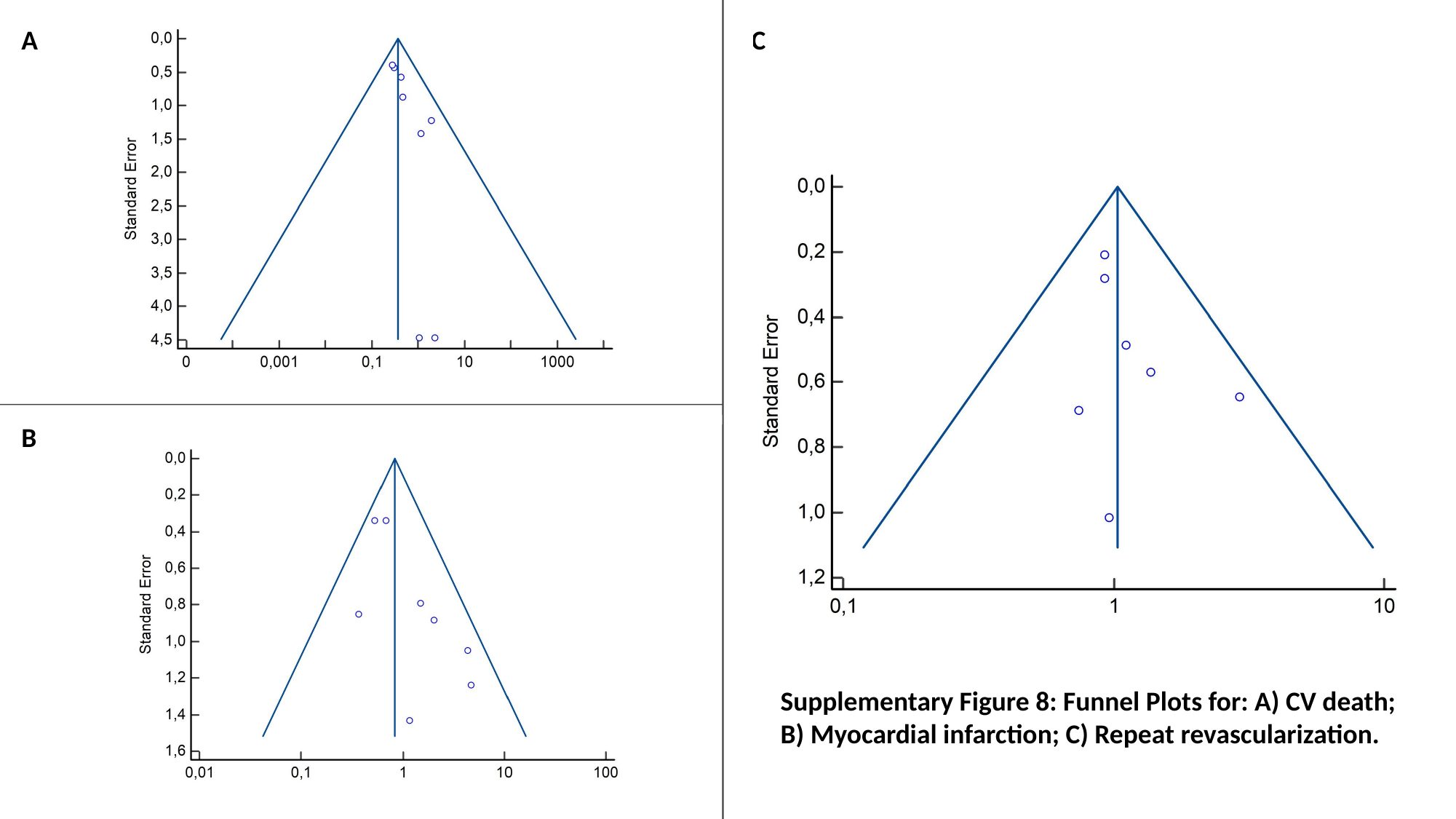

A
C
B
Supplementary Figure 8: Funnel Plots for: A) CV death; B) Myocardial infarction; C) Repeat revascularization.

## Slide 9
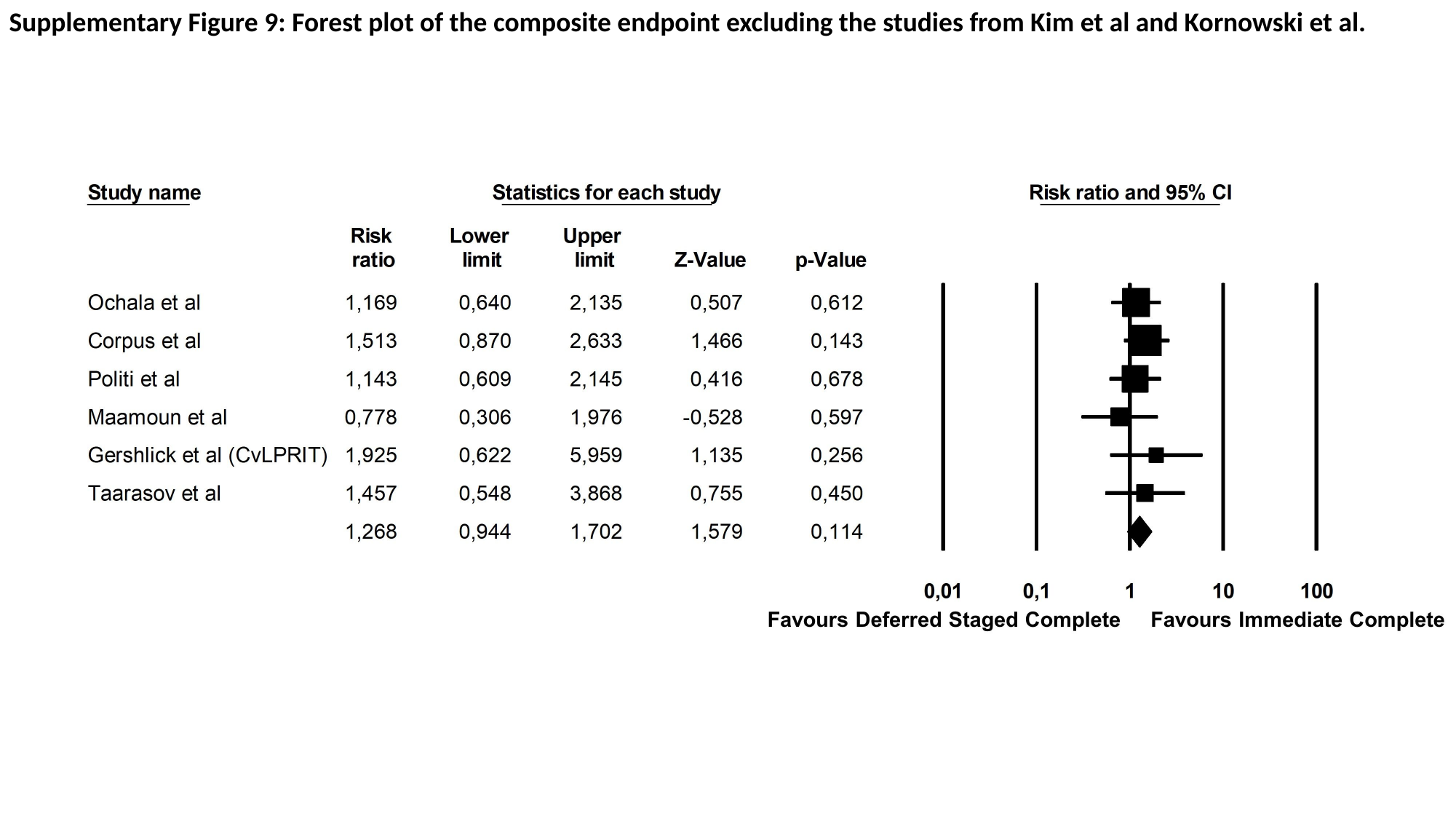

Supplementary Figure 9: Forest plot of the composite endpoint excluding the studies from Kim et al and Kornowski et al.

## Slide 10
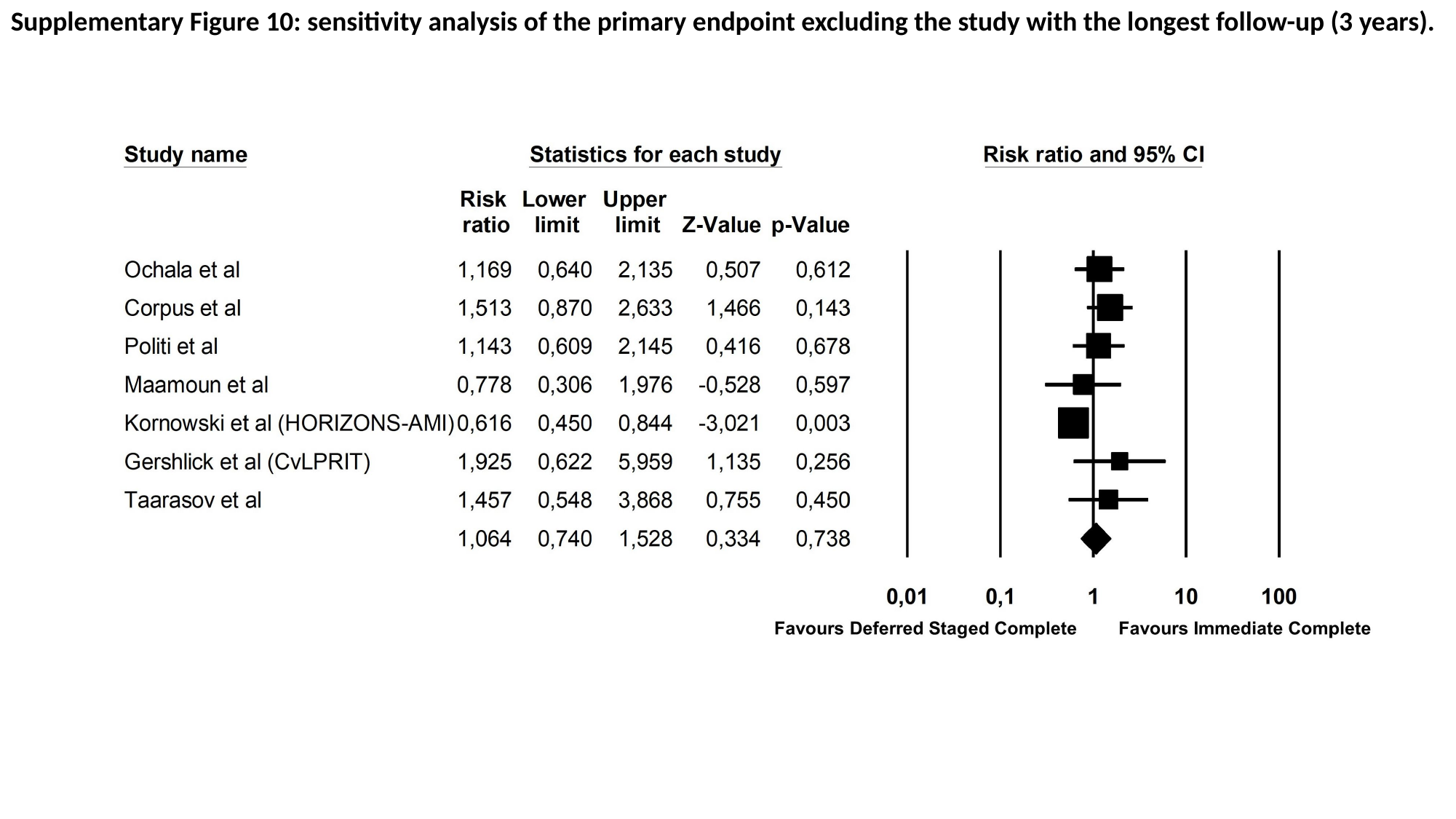

Supplementary Figure 10: sensitivity analysis of the primary endpoint excluding the study with the longest follow-up (3 years).

## Slide 11
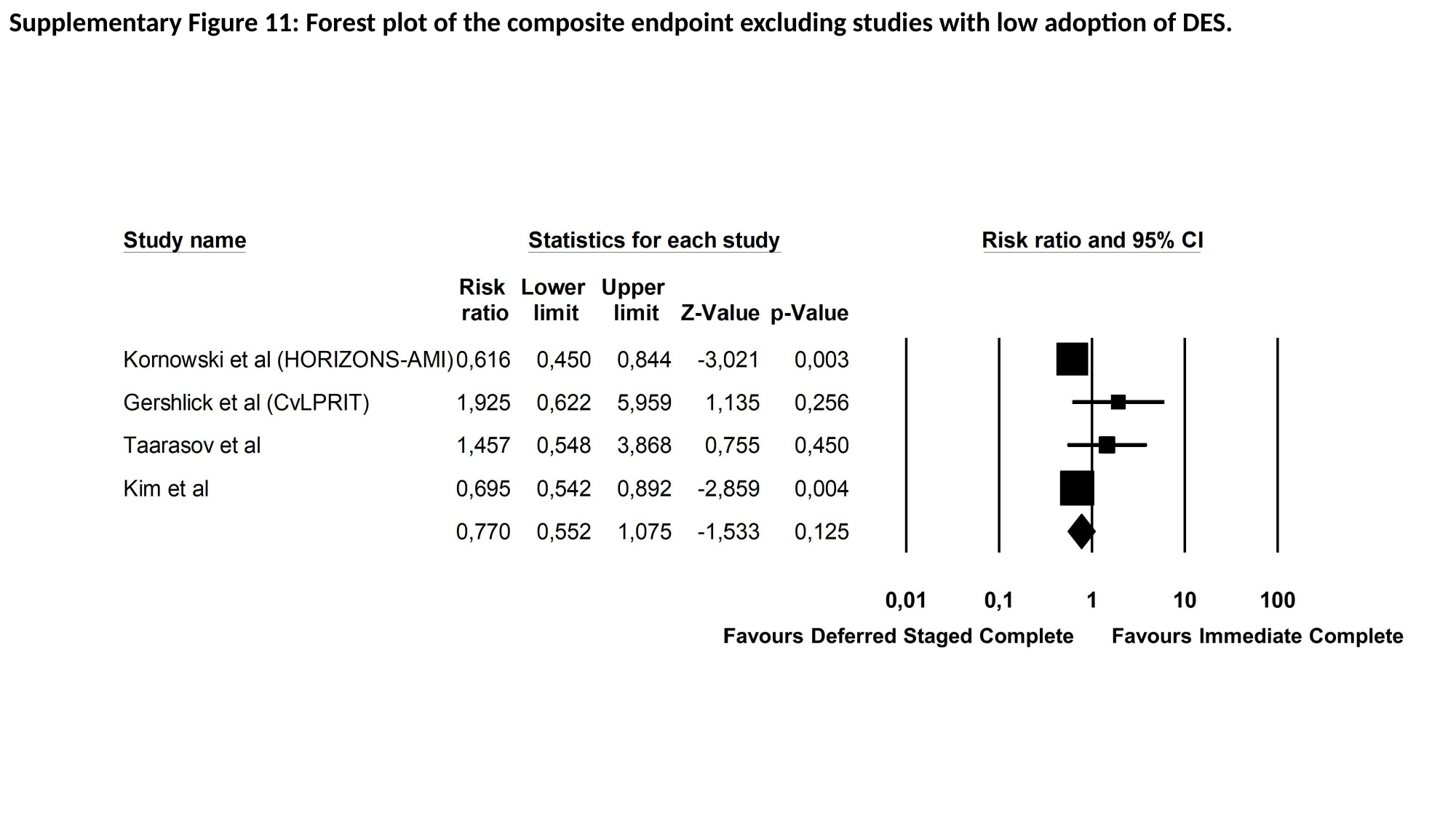

Supplementary Figure 11: Forest plot of the composite endpoint excluding studies with low adoption of DES.

## Slide 12
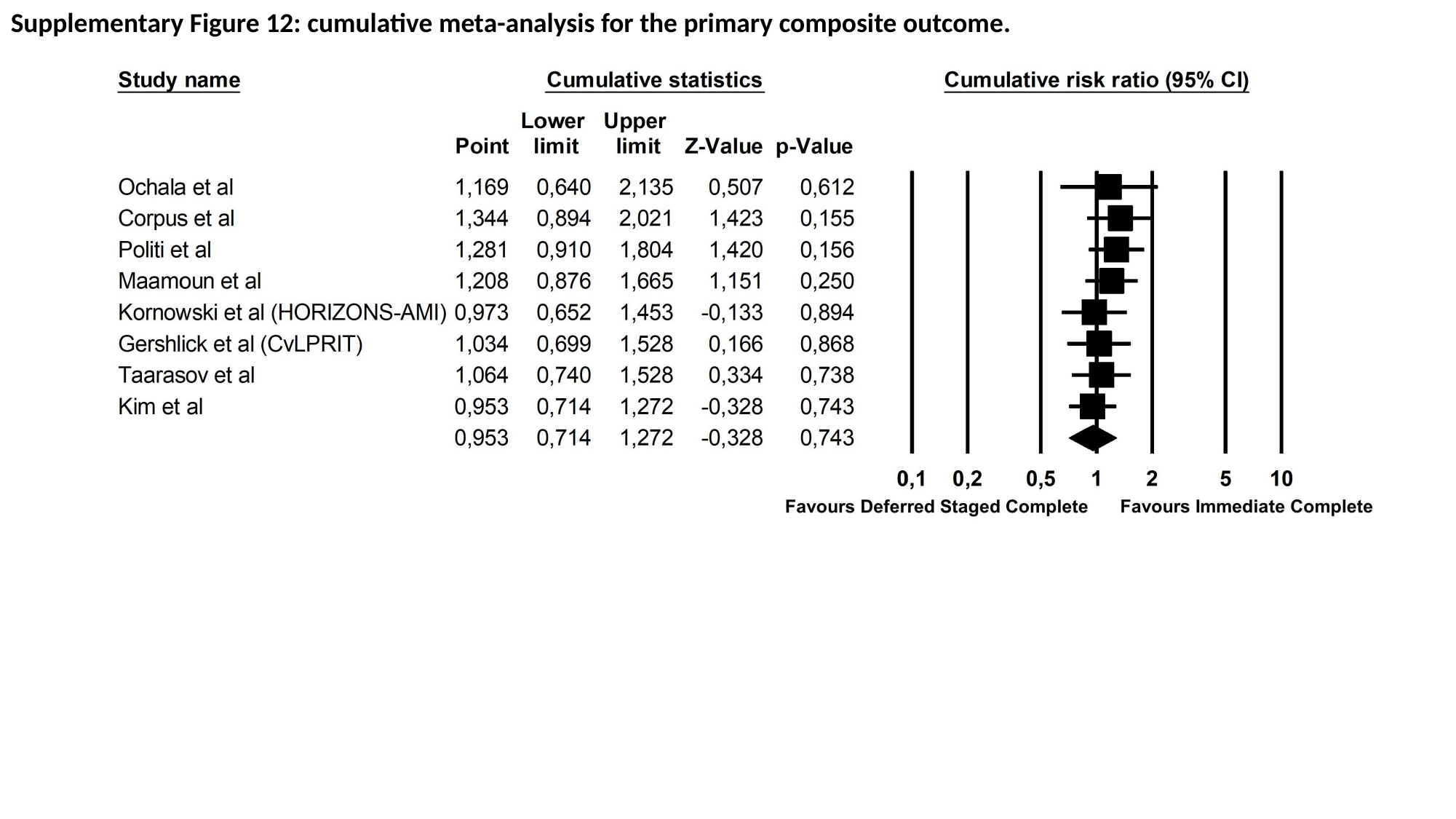

Supplementary Figure 12: cumulative meta-analysis for the primary composite outcome.

## Slide 13
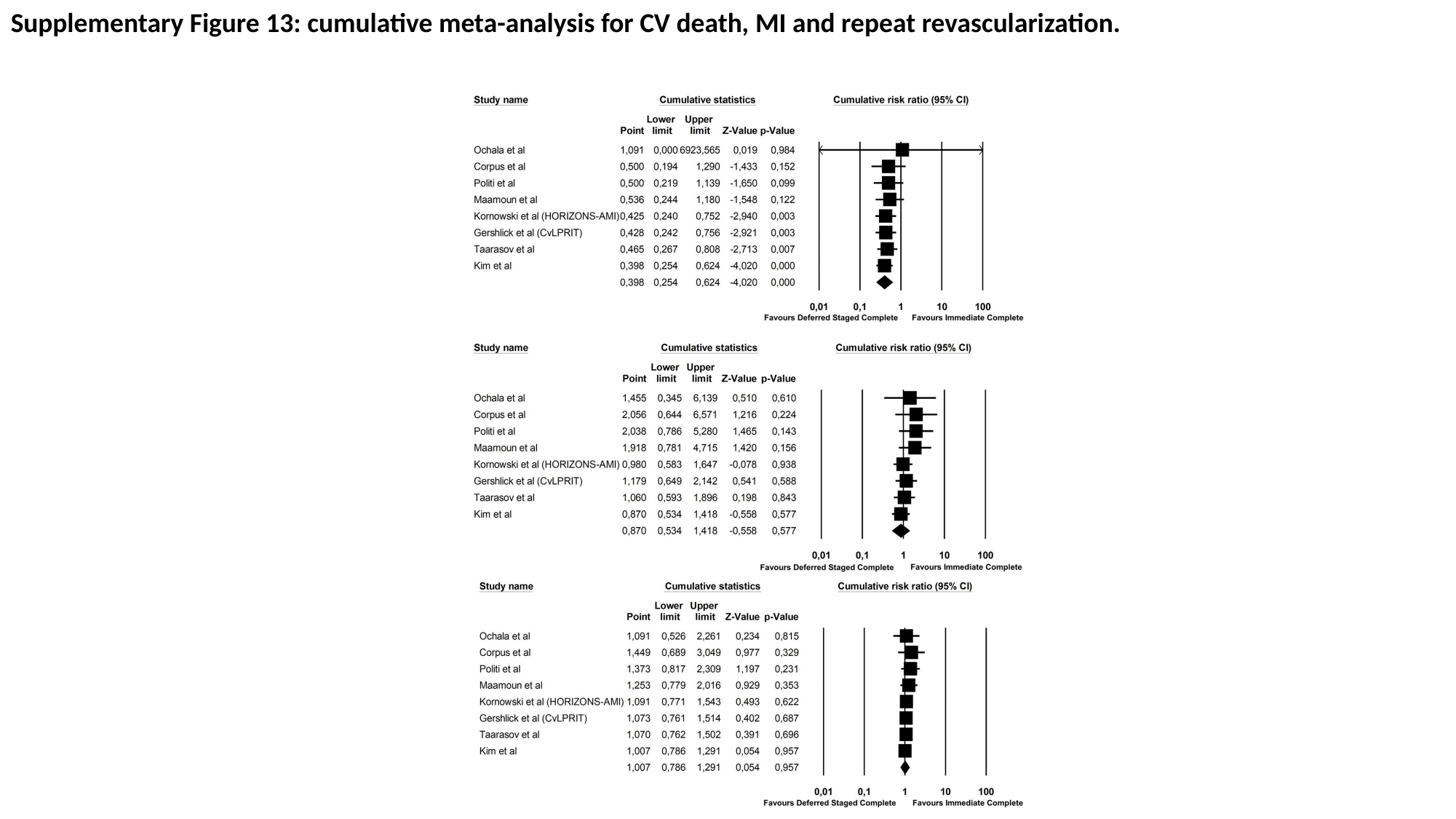

Supplementary Figure 13: cumulative meta-analysis for CV death, MI and repeat revascularization.
