# Supplementary material for: Timing of Complete Revascularization in Patients with STEMI and Multivessel Disease: A Systematic Review and Meta-Analysis
Source: Rev Cardiovasc Med. 2023 Feb 10;24(2):58. doi: 10.31083/j.rcm2402058 (PMC11273107; doi:10.31083/j.rcm2402058)

Supplementary Figure 1: forest plot of CV death (A), MI (B) and repeat revascularization (C) excluding studies with low adoption of DES.

Study name	Risk ratio	Lower limit	Upper limit	Z-Value	p-Value
	**Statistics for each study**				
Kornowski et al (HORIZONS-AMI)	0,329	0,144	0,752	-2,636	0,008
Gershlick et al (CvLPRIT)	2,310	0,000	14688,014	0,187	0,851
Taarasov et al	1,942	0,180	20,916	0,547	0,584
Kim et al	0,295	0,137	0,636	-3,112	0,002
	0,345	0,200	0,595	-3,819	0,000

Risk ratio and 95% CI

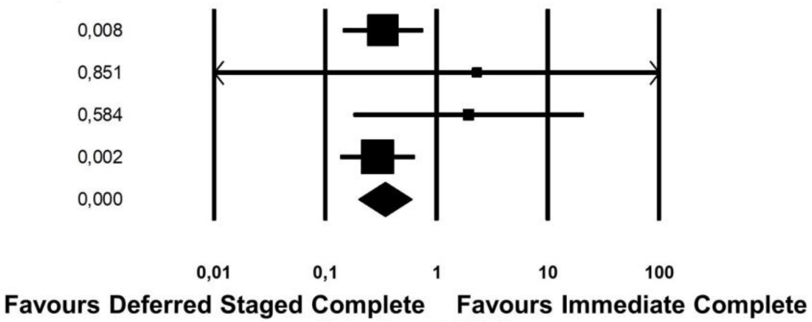

Study name	Risk ratio	Lower limit	Upper limit	Z-Value	p-Value
	**Statistics for each study**				
Kornowski et al (HORIZONS-AMI)	0,700	0,371	1,320	-1,102	0,270
Gershlick et al (CvLPRIT)	4,619	0,430	49,560	1,264	0,206
Taarasov et al	0,388	0,078	1,933	-1,155	0,248
Kim et al	0,551	0,289	1,048	-1,819	0,069
	0,646	0,403	1,036	-1,813	0,070

Risk ratio and 95% CI

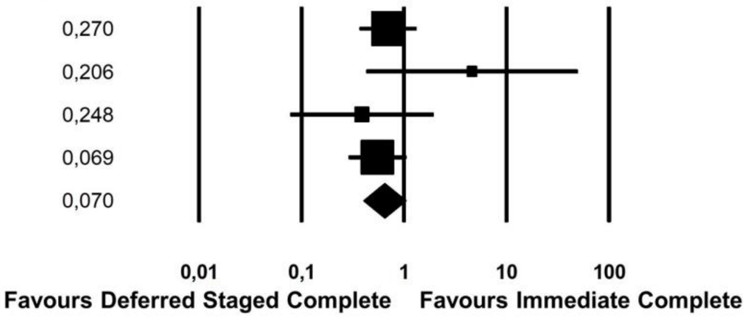

Study name	Risk ratio	Lower limit	Upper limit	Z-Value	p-Value
	**Statistics for each study**				
Kornowski et al (HORIZONS-AMI)	0,933	0,562	1,548	-0,268	0,788
Gershlick et al (CvLPRIT)	0,326	0,017	6,167	-0,748	0,455
Taarasov et al	0,971	0,141	6,696	-0,030	0,976
Kim et al	0,939	0,652	1,351	-0,341	0,733
	0,928	0,694	1,241	-0,505	0,613

Risk ratio and 95% CI

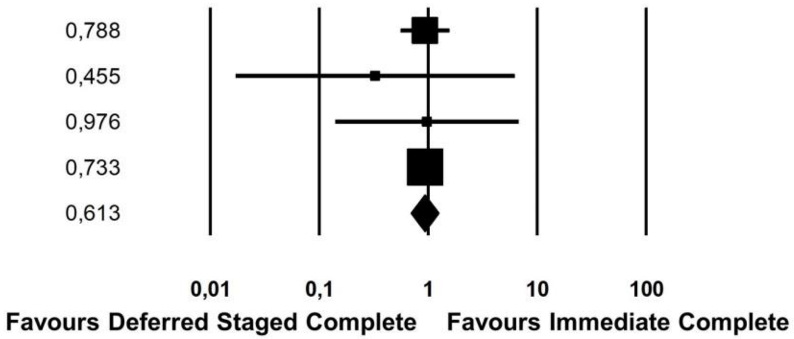

Supplementary Figure 2: Forest plot for acute kidney injury (AKI).

Study name	Risk ratio	Lower limit	Upper limit	Z-Value	p-Value
	Statistics for each study				
Ochala et al	1,091	0,000	6923,565	0,019	0,984
Politi et al	2,000	0,186	21,517	0,572	0,567
Maamoun et al	0,583	0,055	6,171	-0,448	0,654
Taarasov et al	0,971	0,000	6181,787	-0,007	0,995
	1,072	0,213	5,396	0,085	0,933

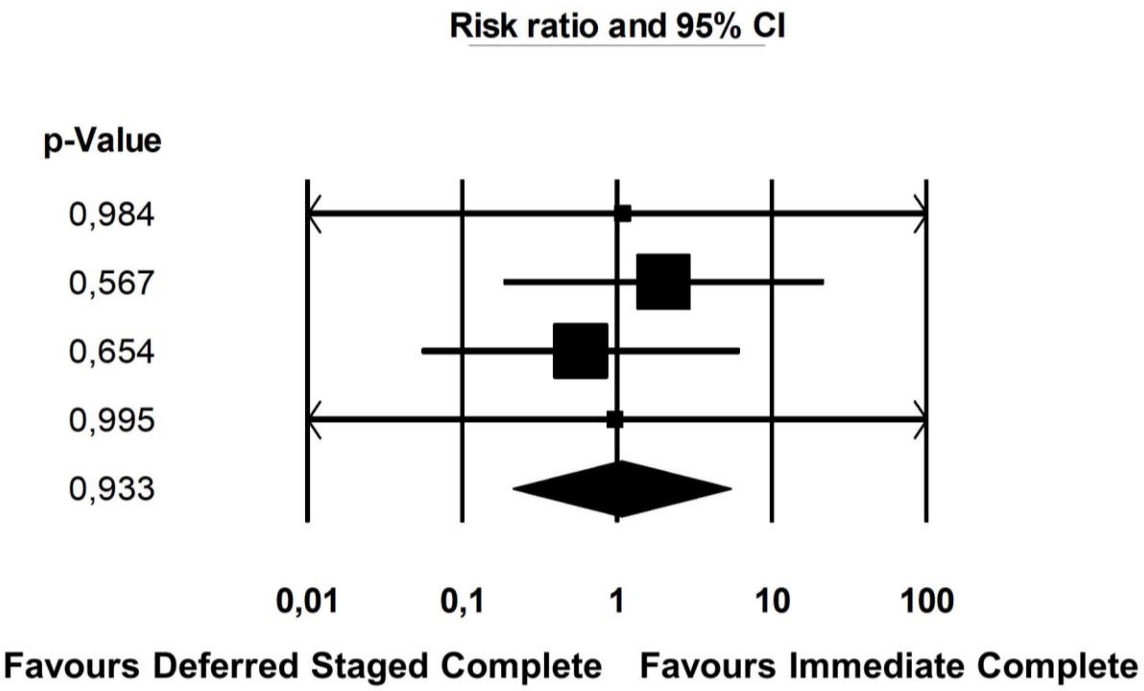

Supplementary Figure 3: Forest plot for trial defined major bleeding.

Study name	Statistics for each study					Risk ratio and 95% CI
	Risk ratio	Lower limit	Upper limit	Z-Value	p-Value	
Corpus et al	1,238	0,156	9,856	0,202	0,840	
Kornowski et al	0,727	0,434	1,217	-1,213	0,225	
	0,750	0,454	1,237	-1,128	0,259	

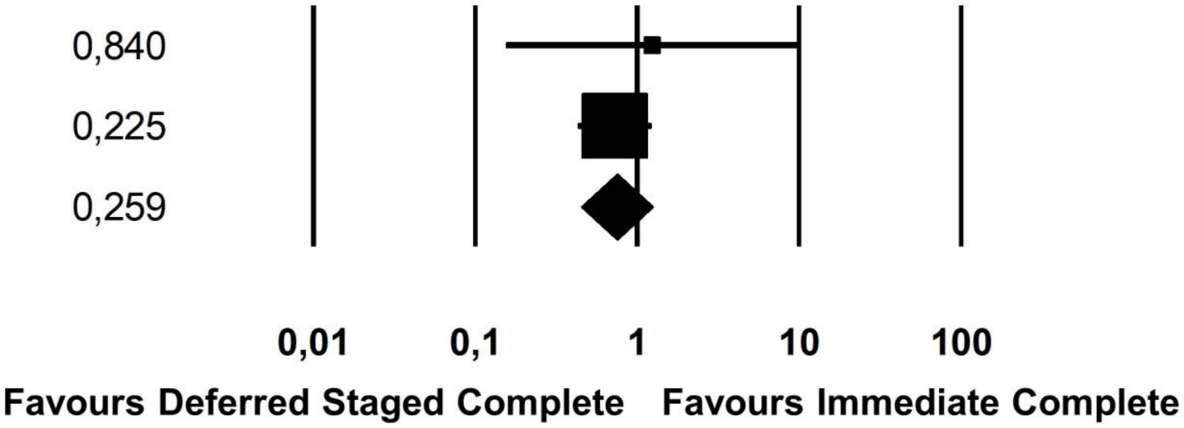

Supplementary Figure 4: traffic light plot for evaluation of bias.

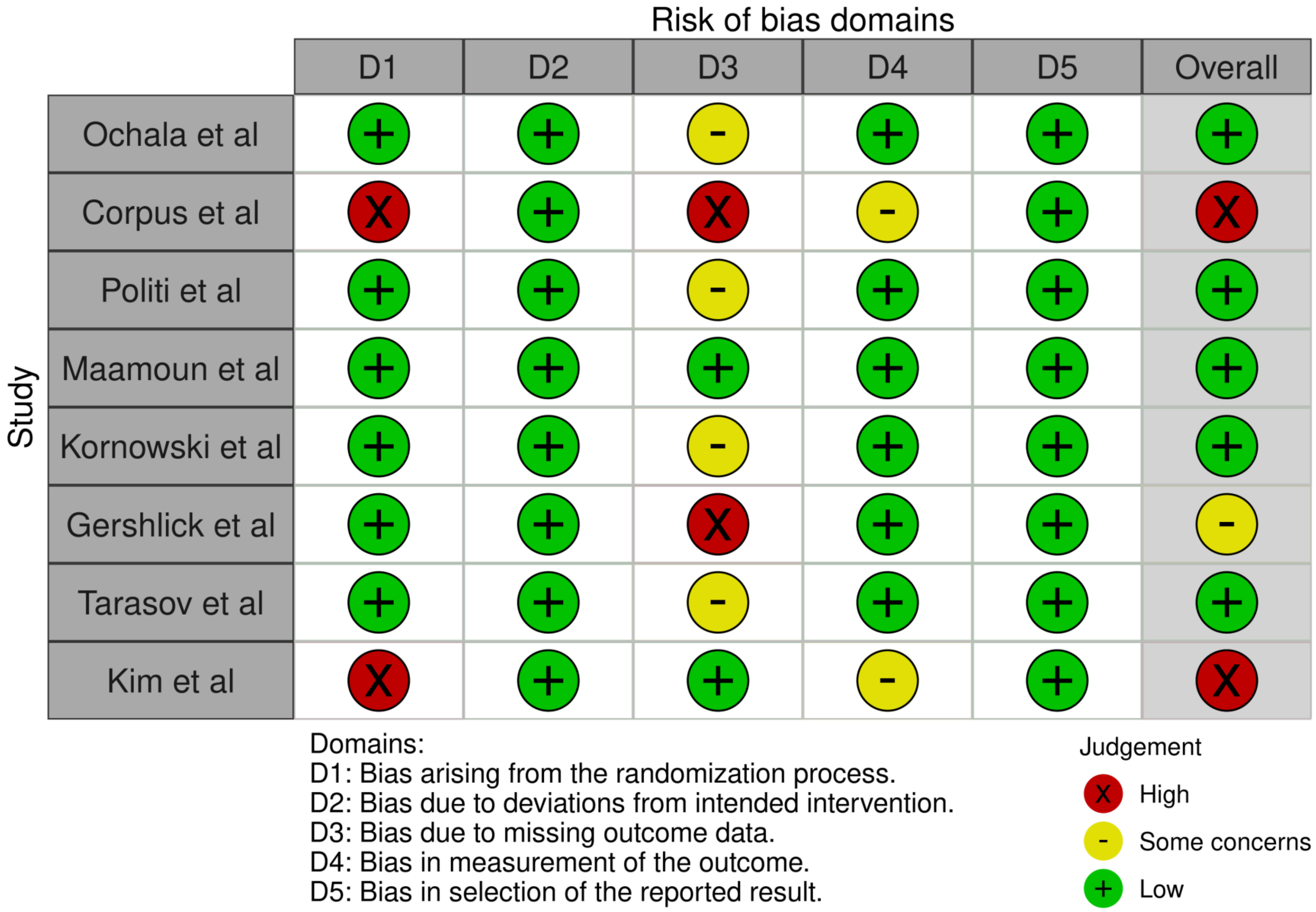

Supplementary Figure 5: Funnel Plots for the primary composite outcome of all cause death, MI and repeat revascularization.

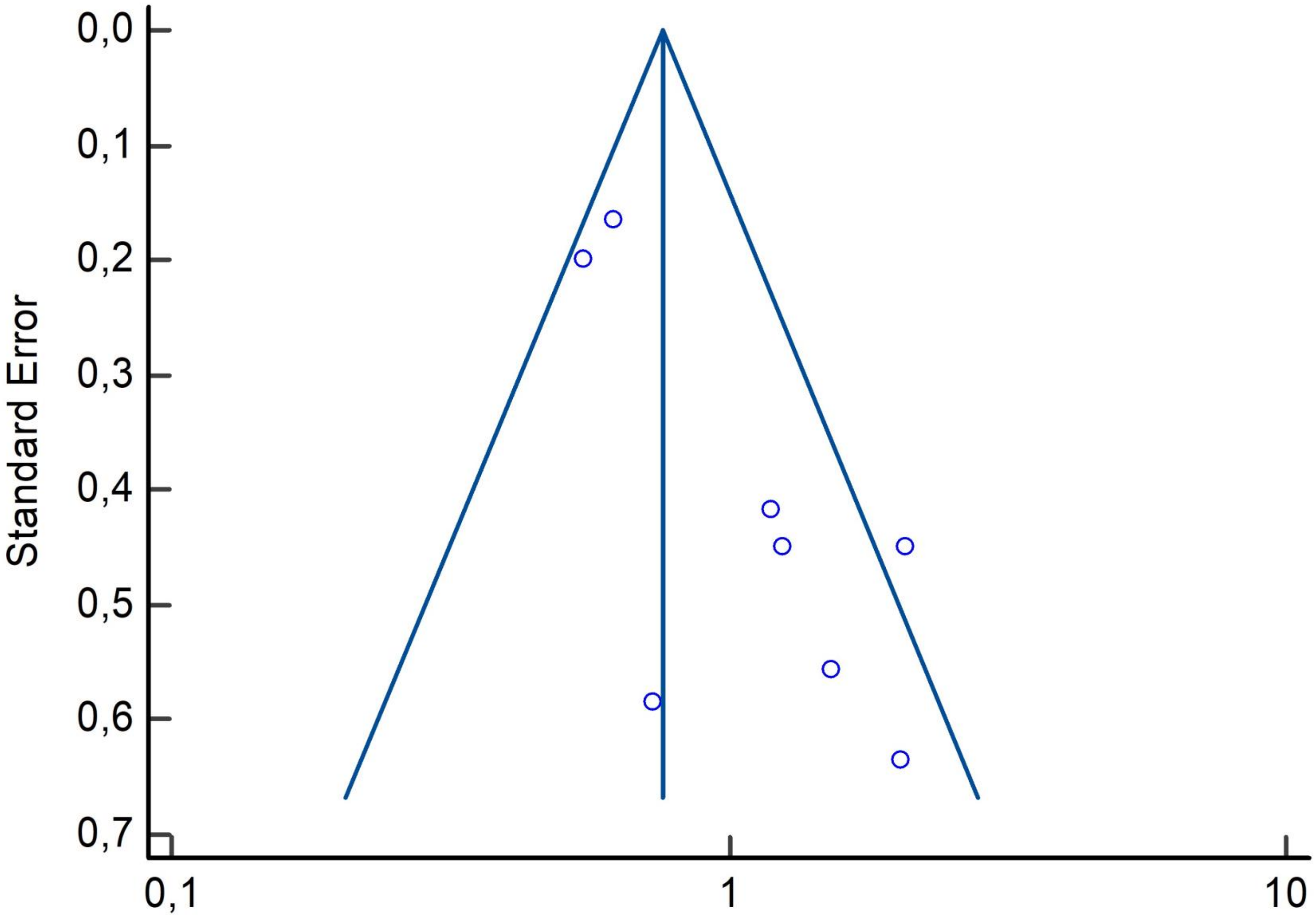

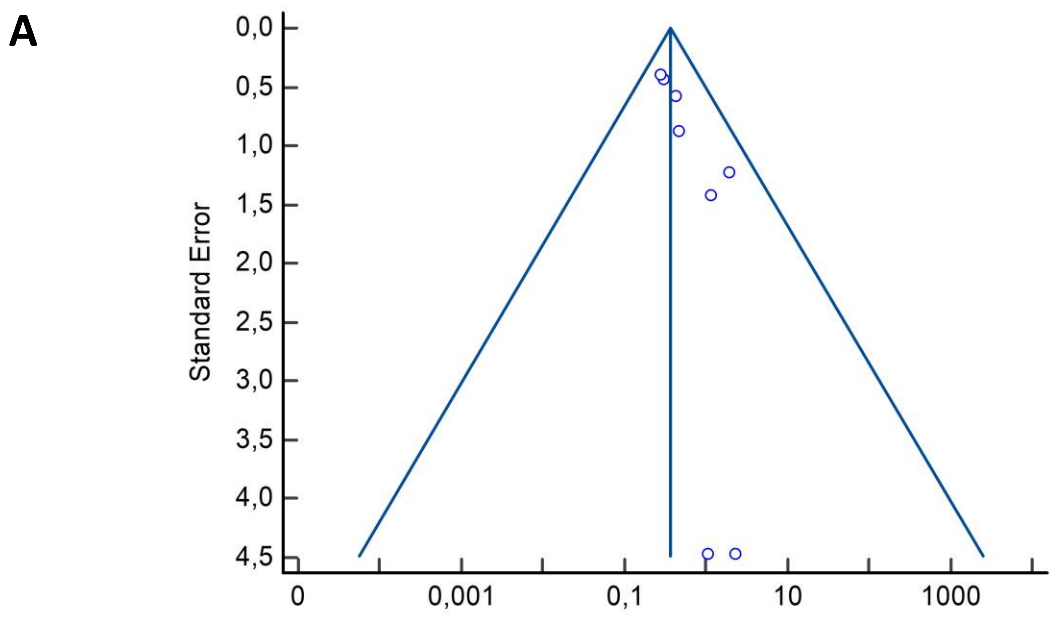

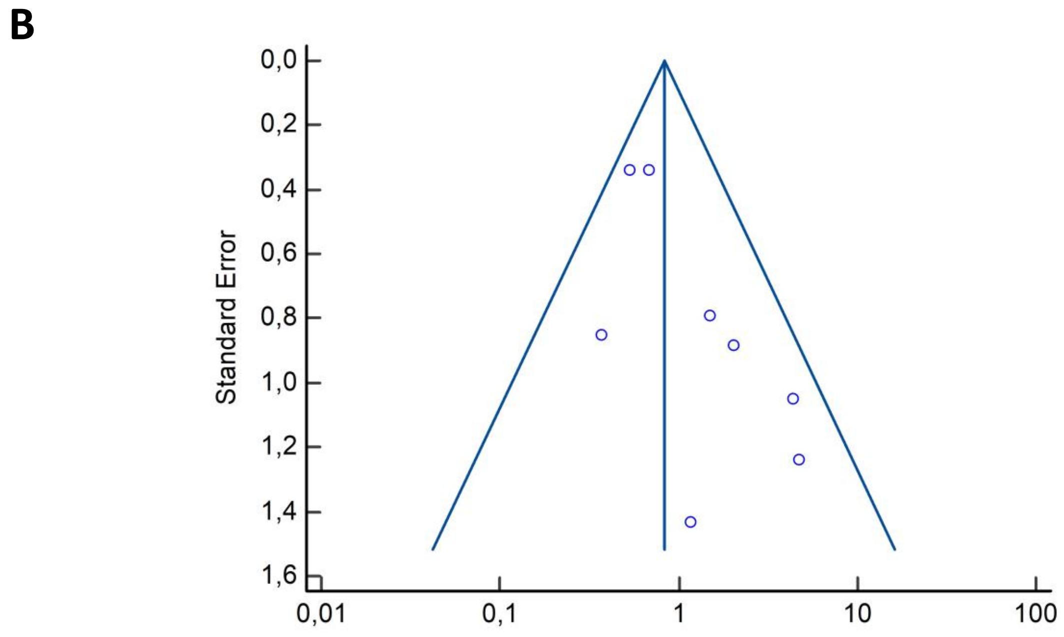

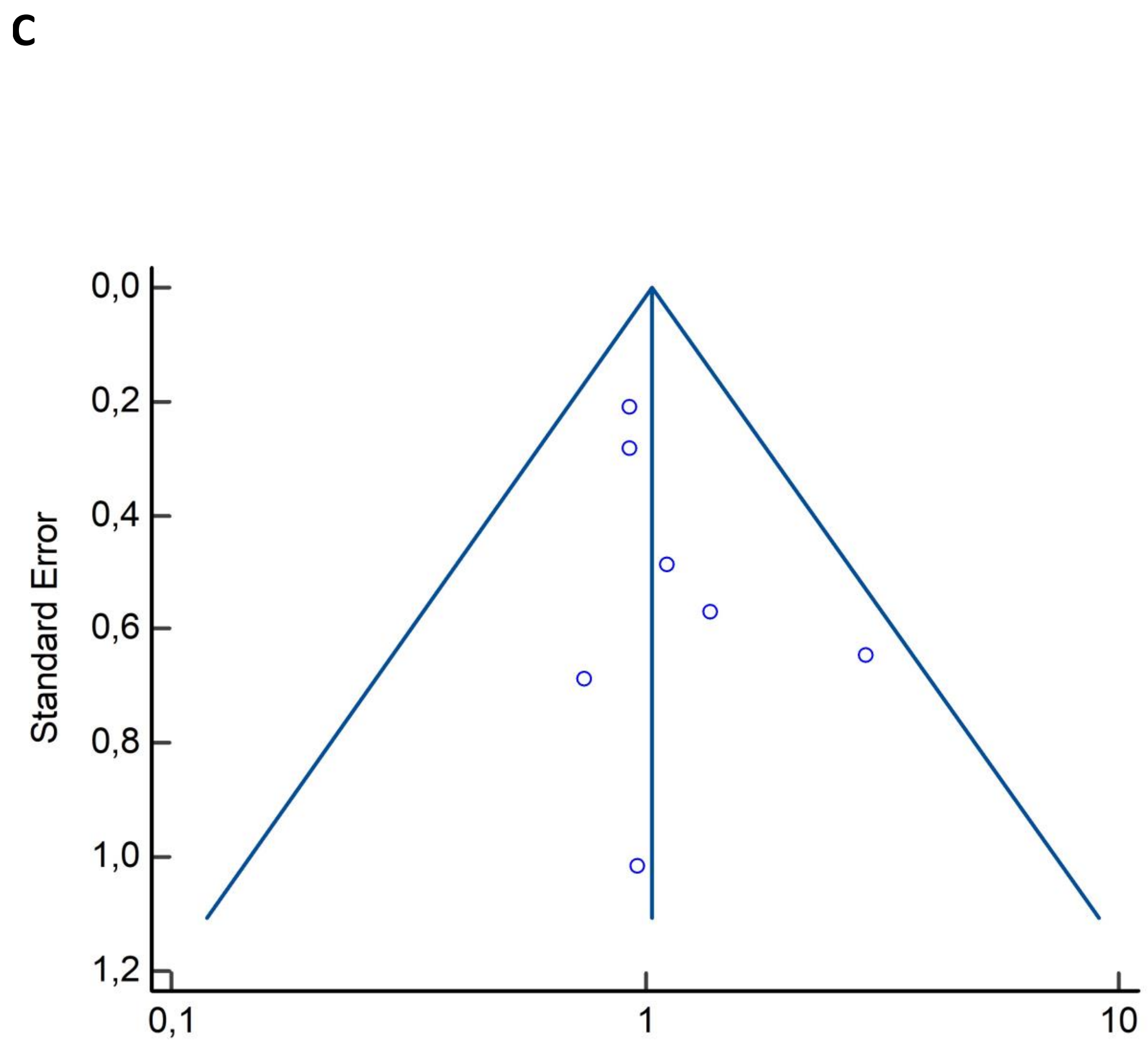

Supplementary Figure 6: Funnel Plots for: A) CV death; B) Myocardial infarction; C) Repeat revascularization.

Supplementary Figure 7: Forest plot of the composite endpoint excluding the studies from Kim et al and Kornowski et al.

Study name	Risk ratio	Lower limit	Upper limit	Z-Value	p-Value
	**Statistics for each study**				
Ochala et al	1,169	0,640	2,135	0,507	0,612
Corpus et al	1,513	0,870	2,633	1,466	0,143
Politi et al	1,143	0,609	2,145	0,416	0,678
Maamoun et al	0,778	0,306	1,976	-0,528	0,597
Gershlick et al (CvLPRIT)	1,925	0,622	5,959	1,135	0,256
Taarasov et al	1,457	0,548	3,868	0,755	0,450
	1,268	0,944	1,702	1,579	0,114

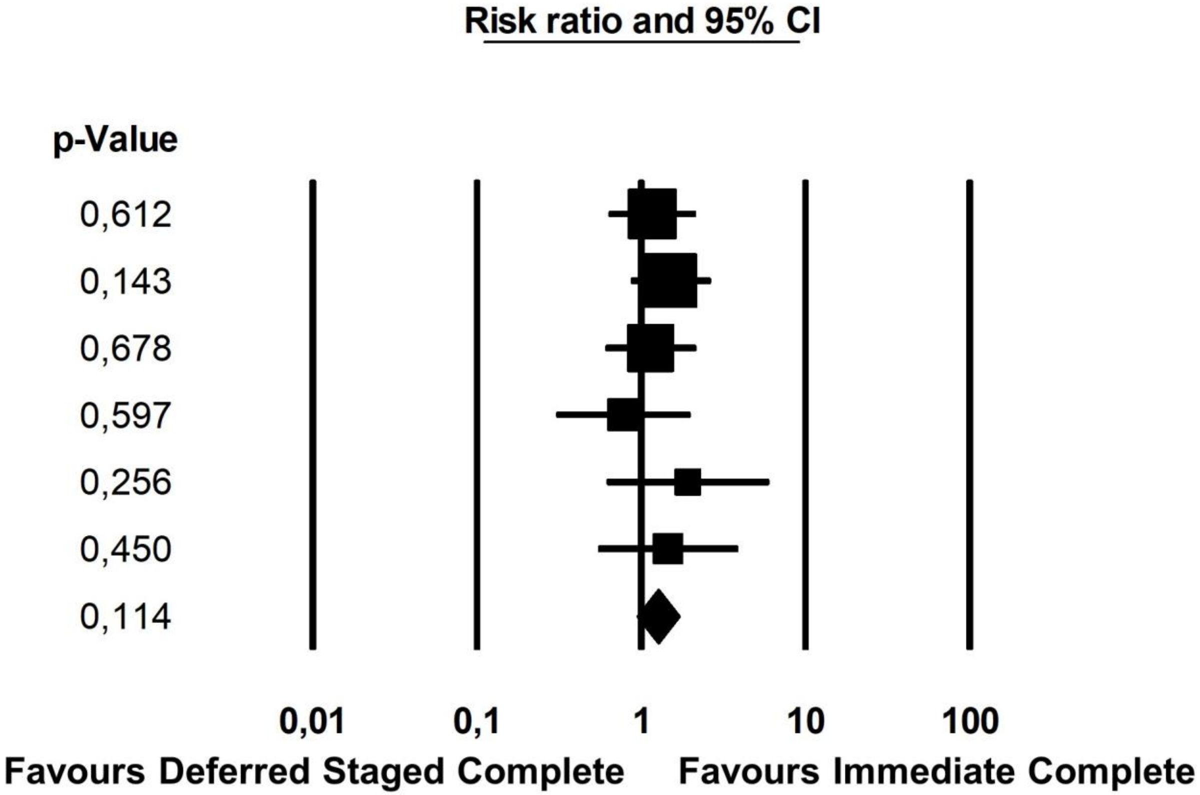

Supplementary Figure 8: Forest plot of the composite endpoint excluding studies with low adoption of DES.

Study name	Risk ratio	Lower limit	Upper limit	Z-Value	p-Value
	Statistics for each study				
Kornowski et al (HORIZONS-AMI)	0,616	0,450	0,844	-3,021	0,003
Gershlick et al (CvLPRIT)	1,925	0,622	5,959	1,135	0,256
Taarasov et al	1,457	0,548	3,868	0,755	0,450
Kim et al	0,695	0,542	0,892	-2,859	0,004
	0,770	0,552	1,075	-1,533	0,125

Risk ratio and 95% CI

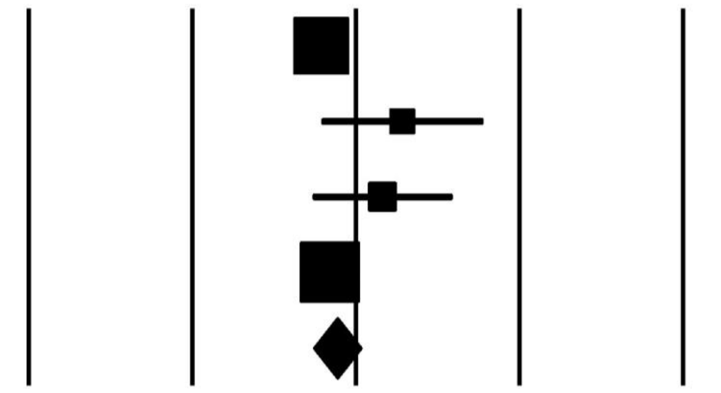

Supplementary Figure 9: cumulative meta-analysis for the primary composite outcome.

Study name	Cumulative statistics				
	Point	Lower limit	Upper limit	Z-Value	p-Value
Ochala et al	1,169	0,640	2,135	0,507	0,612
Corpus et al	1,344	0,894	2,021	1,423	0,155
Politi et al	1,281	0,910	1,804	1,420	0,156
Maamoun et al	1,208	0,876	1,665	1,151	0,250
Kornowski et al (HORIZONS-AMI)	0,973	0,652	1,453	-0,133	0,894
Gershlick et al (CvLPRIT)	1,034	0,699	1,528	0,166	0,868
Taarasov et al	1,064	0,740	1,528	0,334	0,738
Kim et al	0,953	0,714	1,272	-0,328	0,743
	0,953	0,714	1,272	-0,328	0,743

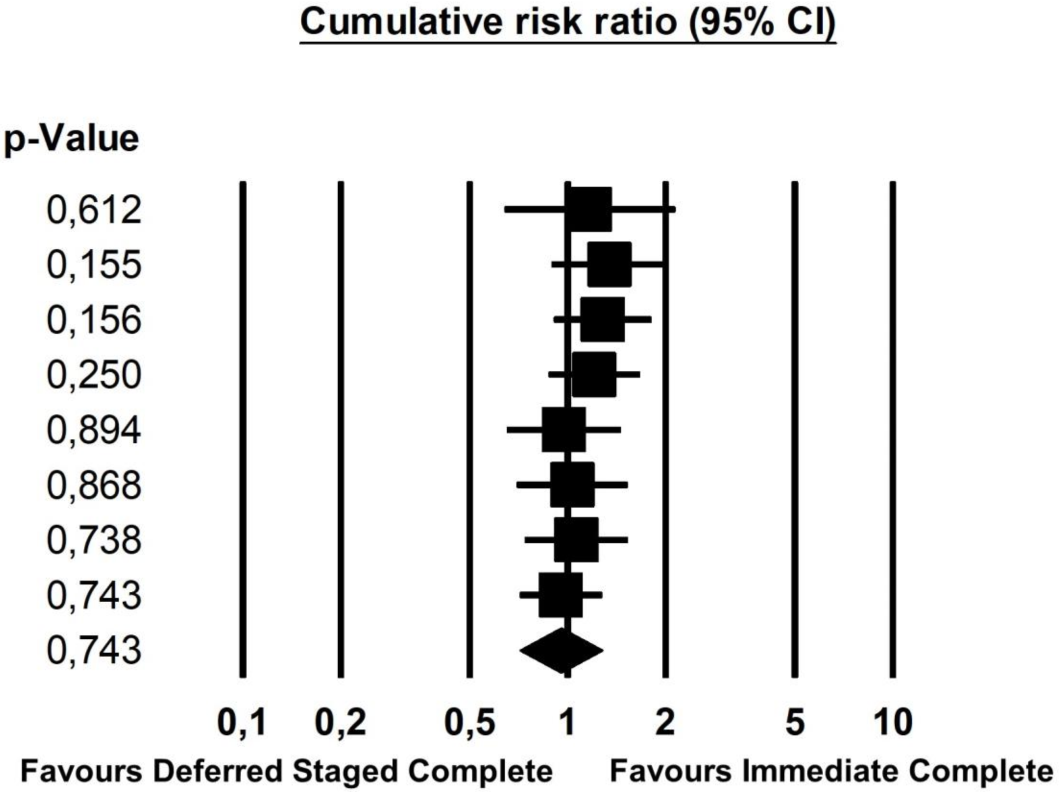

Supplementary Figure 10: cumulative meta-analysis for CV death, MI and repeat revascularization.

Study name	Point	Lower limit	Upper limit	Z-Value	p-Value
Ochala et al	1,091	0,000	6923,565	0,019	0,984
Corpus et al	0,500	0,194	1,290	-1,433	0,152
Politi et al	0,500	0,219	1,139	-1,650	0,099
Maamoun et al	0,536	0,244	1,180	-1,548	0,122
Kornowski et al (HORIZONS-AMI)	0,425	0,240	0,752	-2,940	0,003
Gershlick et al (CvLPRIT)	0,428	0,242	0,756	-2,921	0,003
Taarasov et al	0,465	0,267	0,808	-2,713	0,007
Kim et al	0,398	0,254	0,624	-4,020	0,000
	0,398	0,254	0,624	-4,020	0,000

Cumulative statistics — Cumulative risk ratio (95% CI)

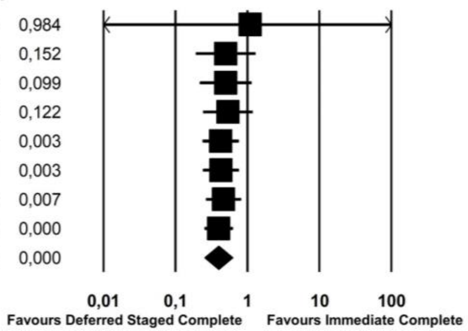

Study name	Point	Lower limit	Upper limit	Z-Value	p-Value
Ochala et al	1,455	0,345	6,139	0,510	0,610
Corpus et al	2,056	0,644	6,571	1,216	0,224
Politi et al	2,038	0,786	5,280	1,465	0,143
Maamoun et al	1,918	0,781	4,715	1,420	0,156
Kornowski et al (HORIZONS-AMI)	0,980	0,583	1,647	-0,078	0,938
Gershlick et al (CvLPRIT)	1,179	0,649	2,142	0,541	0,588
Taarasov et al	1,060	0,593	1,896	0,198	0,843
Kim et al	0,870	0,534	1,418	-0,558	0,577
	0,870	0,534	1,418	-0,558	0,577

Cumulative statistics — Cumulative risk ratio (95% CI)

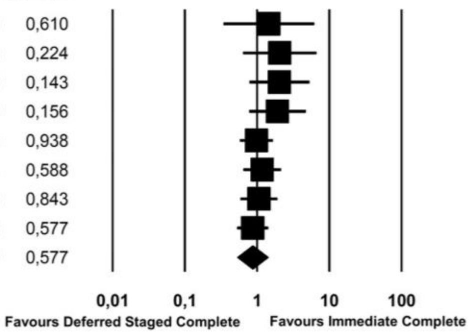

Study name	Point	Lower limit	Upper limit	Z-Value	p-Value
Ochala et al	1,091	0,526	2,261	0,234	0,815
Corpus et al	1,449	0,689	3,049	0,977	0,329
Politi et al	1,373	0,817	2,309	1,197	0,231
Maamoun et al	1,253	0,779	2,016	0,929	0,353
Kornowski et al (HORIZONS-AMI)	1,091	0,771	1,543	0,493	0,622
Gershlick et al (CvLPRIT)	1,073	0,761	1,514	0,402	0,687
Taarasov et al	1,070	0,762	1,502	0,391	0,696
Kim et al	1,007	0,786	1,291	0,054	0,957
	1,007	0,786	1,291	0,054	0,957

Cumulative statistics — Cumulative risk ratio (95% CI)

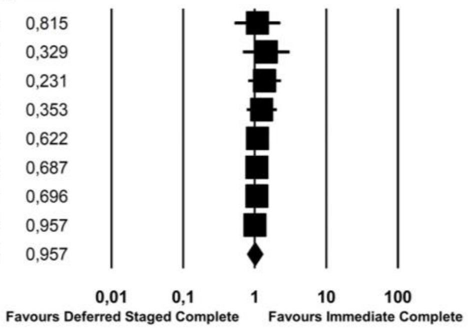

Supplementary Figure 11: meta regression of the effect of time to staged PCI on the primary endpoint.

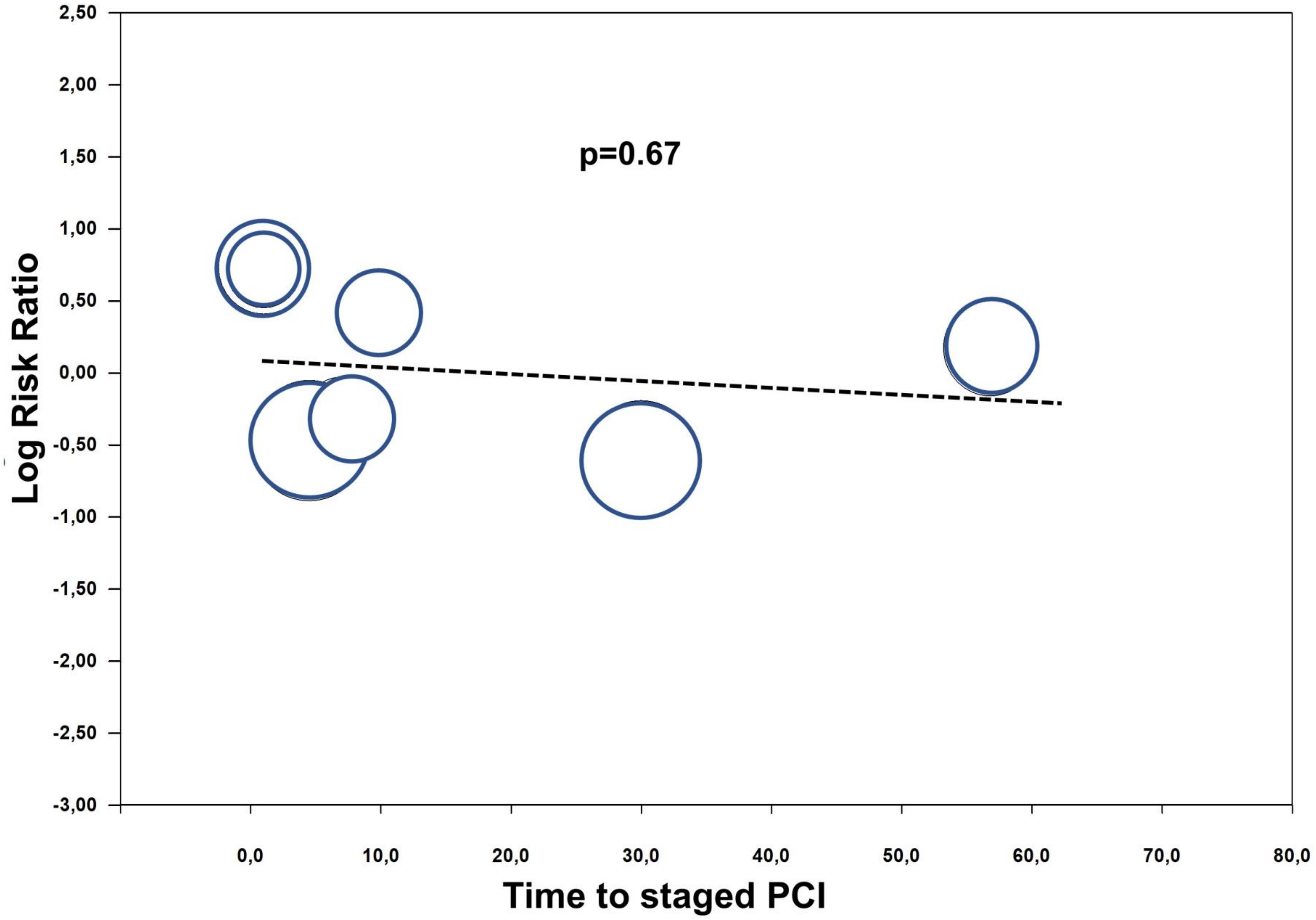

Supplementary Figure 12: meta regression of the primary endpoint using infarct location on the anterior wall as the moderator.

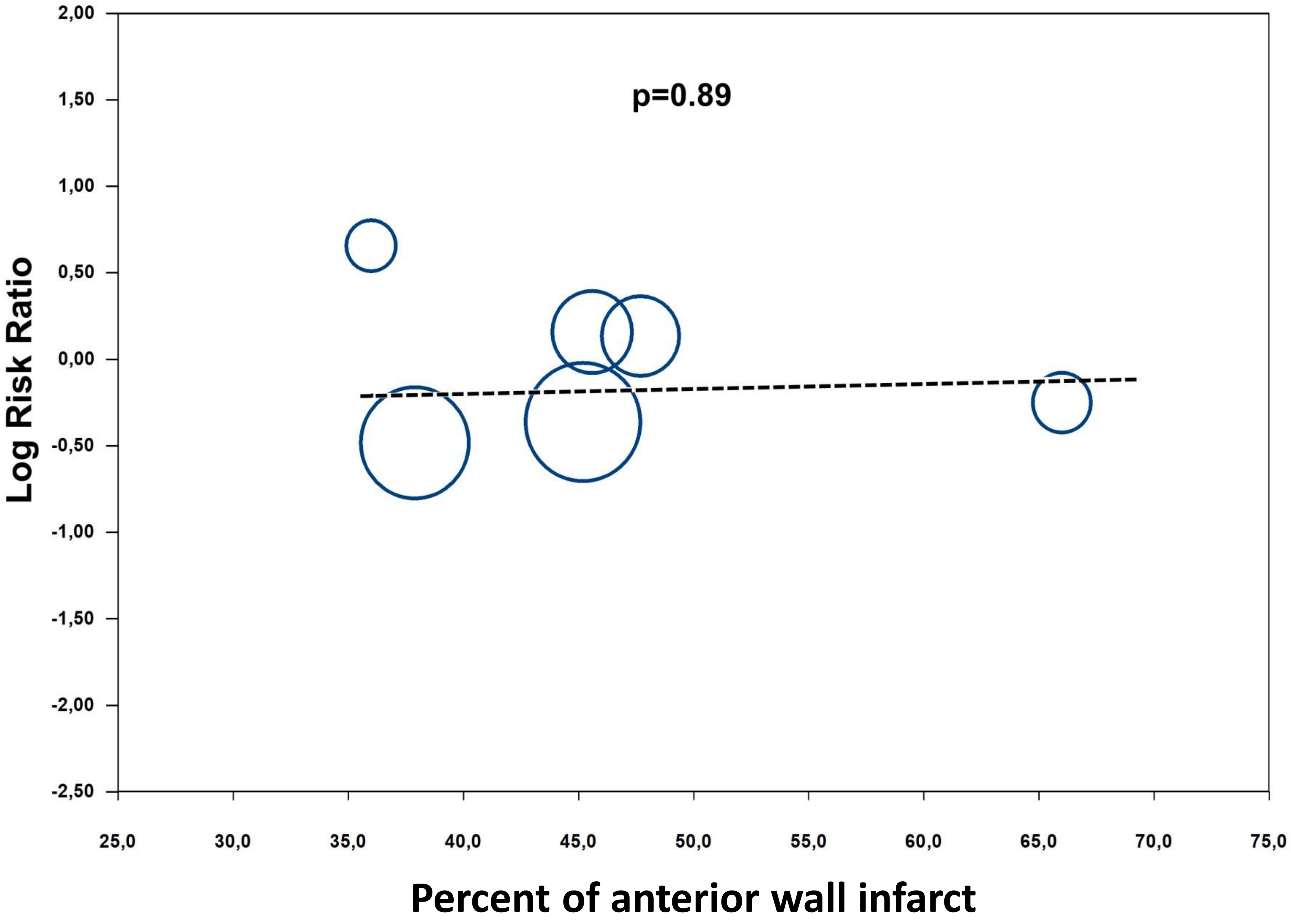

Supplementary Figure 13: sensitivity analysis of the primary endpoint excluding the study with the longest follow-up (3 years).

Study name	Risk ratio	Lower limit	Upper limit	Z-Value	p-Value
Ochala et al	1,169	0,640	2,135	0,507	0,612
Corpus et al	1,513	0,870	2,633	1,466	0,143
Politi et al	1,143	0,609	2,145	0,416	0,678
Maamoun et al	0,778	0,306	1,976	-0,528	0,597
Kornowski et al (HORIZONS-AMI)	0,616	0,450	0,844	-3,021	0,003
Gershlick et al (CvLPRIT)	1,925	0,622	5,959	1,135	0,256
Taarasov et al	1,457	0,548	3,868	0,755	0,450
	1,064	0,740	1,528	0,334	0,738

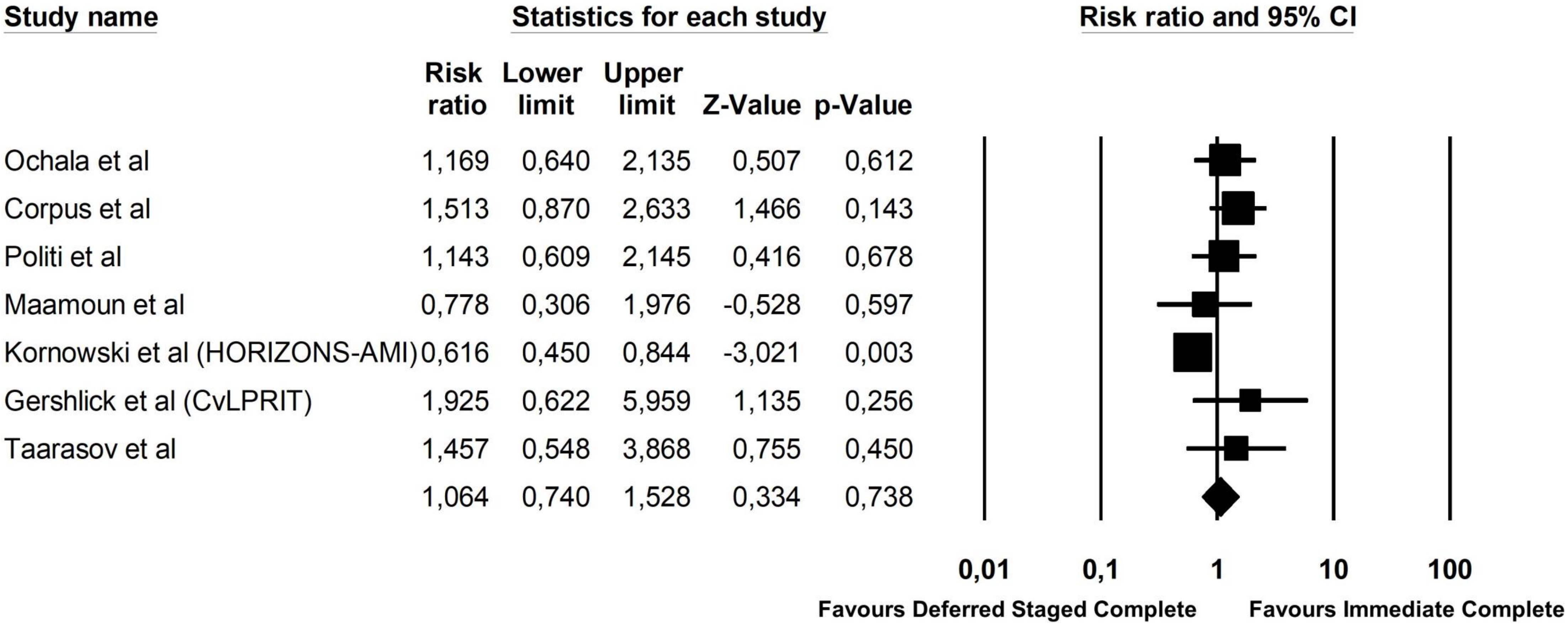

Supplement: Supplementary file 1 [file 2153-8174-24-2-058-s1.zip › Supplementary File.pdf]
